# Differential cell signaling testing for cell-cell communication inference from single-cell data by dominoSignal

**DOI:** 10.1093/bioinformatics/btag089

**Published:** 2026-02-26

**Authors:** Jacob T Mitchell, Orian Stapleton, Kavita Krishnan, Sushma Nagaraj, Dmitrijs Lvovs, Christopher Cherry, Amanda Poissonnier, Wesley Horton, Andrew Adey, Varun Rao, Amanda Huff, Jacquelyn W Zimmerman, Luciane T Kagohara, Neeha Zaidi, Lisa M Coussens, Elizabeth M Jaffee, Jennifer H Elisseeff, Elana J Fertig

**Affiliations:** Department of Oncology, Sidney Kimmel Comprehensive Cancer Center, Johns Hopkins University, Baltimore, MD 21205, United States; Johns Hopkins Convergence Institute, Johns Hopkins University, Baltimore, MD 21205, United States; Johns Hopkins Bloomberg Kimmel Institute, Johns Hopkins University, Baltimore, MD 21205, United States; Quantitative Sciences Division, Department of Oncology, Johns Hopkins University, Baltimore, MD 21205, United States; Department of Genetic Medicine, Johns Hopkins University, Baltimore, MD 21205, United States; Department of Oncology, Sidney Kimmel Comprehensive Cancer Center, Johns Hopkins University, Baltimore, MD 21205, United States; Johns Hopkins Convergence Institute, Johns Hopkins University, Baltimore, MD 21205, United States; Johns Hopkins Bloomberg Kimmel Institute, Johns Hopkins University, Baltimore, MD 21205, United States; Quantitative Sciences Division, Department of Oncology, Johns Hopkins University, Baltimore, MD 21205, United States; Department of Biomedical Engineering, Johns Hopkins University, Baltimore, MD 21218, United States; Department of Biomedical Engineering, Johns Hopkins University, Baltimore, MD 21218, United States; Translational Tissue Engineering Center, Johns Hopkins University, Baltimore, MD 21231, United States; Institute for Genome Sciences, University of Maryland School of Medicine, Baltimore, MD 21201, United States; Department of Oncology, Sidney Kimmel Comprehensive Cancer Center, Johns Hopkins University, Baltimore, MD 21205, United States; Quantitative Sciences Division, Department of Oncology, Johns Hopkins University, Baltimore, MD 21205, United States; Institute for Genome Sciences, University of Maryland School of Medicine, Baltimore, MD 21201, United States; Institute for Genome Sciences, University of Maryland School of Medicine, Baltimore, MD 21201, United States; Department of Medicine, University of Maryland School of Medicine, Baltimore, MD 21201, United States; Department of Biomedical Engineering, Johns Hopkins University, Baltimore, MD 21218, United States; Translational Tissue Engineering Center, Johns Hopkins University, Baltimore, MD 21231, United States; Department of Cell, Developmental & Cancer Biology, Oregon Health & Science University, Portland, OR 97239, United States; Department of Cell, Developmental & Cancer Biology, Oregon Health & Science University, Portland, OR 97239, United States; Department of Molecular & Medical Genetics, Oregon Health & Science University, Portland, OR 97239, United States; Knight Cancer Institute, Oregon Health & Science University, Portland, OR 97239, United States; Institute for Genome Sciences, University of Maryland School of Medicine, Baltimore, MD 21201, United States; Department of Medicine, University of Maryland School of Medicine, Baltimore, MD 21201, United States; Department of Oncology, Sidney Kimmel Comprehensive Cancer Center, Johns Hopkins University, Baltimore, MD 21205, United States; Johns Hopkins Convergence Institute, Johns Hopkins University, Baltimore, MD 21205, United States; Johns Hopkins Bloomberg Kimmel Institute, Johns Hopkins University, Baltimore, MD 21205, United States; Department of Oncology, Sidney Kimmel Comprehensive Cancer Center, Johns Hopkins University, Baltimore, MD 21205, United States; Johns Hopkins Convergence Institute, Johns Hopkins University, Baltimore, MD 21205, United States; Johns Hopkins Bloomberg Kimmel Institute, Johns Hopkins University, Baltimore, MD 21205, United States; Department of Oncology, Sidney Kimmel Comprehensive Cancer Center, Johns Hopkins University, Baltimore, MD 21205, United States; Johns Hopkins Convergence Institute, Johns Hopkins University, Baltimore, MD 21205, United States; Johns Hopkins Bloomberg Kimmel Institute, Johns Hopkins University, Baltimore, MD 21205, United States; Department of Oncology, Sidney Kimmel Comprehensive Cancer Center, Johns Hopkins University, Baltimore, MD 21205, United States; Johns Hopkins Convergence Institute, Johns Hopkins University, Baltimore, MD 21205, United States; Johns Hopkins Bloomberg Kimmel Institute, Johns Hopkins University, Baltimore, MD 21205, United States; Department of Cell, Developmental & Cancer Biology, Oregon Health & Science University, Portland, OR 97239, United States; Knight Cancer Institute, Oregon Health & Science University, Portland, OR 97239, United States; Department of Oncology, Sidney Kimmel Comprehensive Cancer Center, Johns Hopkins University, Baltimore, MD 21205, United States; Johns Hopkins Convergence Institute, Johns Hopkins University, Baltimore, MD 21205, United States; Johns Hopkins Bloomberg Kimmel Institute, Johns Hopkins University, Baltimore, MD 21205, United States; Department of Biomedical Engineering, Johns Hopkins University, Baltimore, MD 21218, United States; Translational Tissue Engineering Center, Johns Hopkins University, Baltimore, MD 21231, United States; Department of Chemical and Biomedical Engineering, Johns Hopkins University, Baltimore, MD 21218, United States; Department of Oncology, Sidney Kimmel Comprehensive Cancer Center, Johns Hopkins University, Baltimore, MD 21205, United States; Johns Hopkins Convergence Institute, Johns Hopkins University, Baltimore, MD 21205, United States; Johns Hopkins Bloomberg Kimmel Institute, Johns Hopkins University, Baltimore, MD 21205, United States; Quantitative Sciences Division, Department of Oncology, Johns Hopkins University, Baltimore, MD 21205, United States; Department of Biomedical Engineering, Johns Hopkins University, Baltimore, MD 21218, United States; Institute for Genome Sciences, University of Maryland School of Medicine, Baltimore, MD 21201, United States; Department of Medicine, University of Maryland School of Medicine, Baltimore, MD 21201, United States; Department of Applied Mathematics & Statistics, Whiting School of Engineering, Johns Hopkins University, Baltimore, MD 21218, United States; Greenebaum Comprehensive Cancer Center, University of Maryland School of Medicine, Baltimore, MD 21201, United States

## Abstract

**Motivation:**

Algorithms for ligand-receptor network inference have emerged as commonly used tools to estimate cell-cell communication from reference single-cell data. Many studies employ these algorithms to compare signaling between conditions and lack methods to statistically identify signals that are significantly different. We previously developed the cell communication inference algorithm Domino, which considers ligand and receptor gene expression in association with downstream transcription factor activity scoring. We developed the dominoSignal software to innovate upon Domino and extend its functionality to test statistically differential cellular signaling.

**Results:**

This new functionality includes the compilation of active signals as linkages from multiple subjects in a single-cell data set and testing condition-dependent signaling linkage. The software is applicable for analysis of single-cell data sets with multiple subjects as biological replicates as well as with bootstrapped replicates from data sets with few or pooled subjects. We use simulation studies to benchmark the number of subjects in compared groups and cells within an annotated cell type sufficient to accurately identify differential linkages. We demonstrate the application of the Differential Cell Signaling Test (DCST) in the dominoSignal software to investigate consequences of cancer cell phenotypes and immunotherapy on cell-cell communication in tumor microenvironments. These applications in cancer studies demonstrate the ability of differential cell signaling analysis to infer changes to cell communication networks from therapeutic or experimental perturbations, which is broadly applicable across biological systems.

**Availability:**

dominoSignal is available through Bioconductor at https://www.bioconductor.org/packages/release/bioc/html/dominoSignal.html

## 1 Introduction

Many algorithms for Cell-Cell Communication Inference (CCCI) (Armingol *et al.* 2021, Luo *et al.* 2023) have been developed to infer how multicellular systems coordinate development and responses to stimuli via expression of genes encoding ligands and receptors from single-cell RNA-seq (scRNA-seq) datasets. Most CCCI methods infer communication between cell types based on co-expression of ligands by sender cells capable of activating receptors expressed by recipient cells. Methods that derive a signaling score as the product of ligand and receptor gene expression by interacting cell types showed poor accuracy in constructing signaling networks when applied to data sets with orthogonal quantification of signaling interactions (Luo *et al.* 2023). One source of the inaccuracy of these estimates may rise from the failure to consider the expression of other genes encoding components of multi-subunit ligands or receptors. More advanced methods have sought to improve accuracy by accounting for the expression of signaling co-factors or measures of intracellular signaling cascades initiated following receptor activation such as target gene transcription (Choi *et al.* 2015, Wang *et al.* 2019, Baccin *et al.* 2020, Browaeys *et al.* 2020, Efremova *et al.* 2020, Cheng *et al.* 2021, Hu *et al.* 2021, Jin *et al.* 2021, Li *et al.* 2023, Wilk *et al.* 2024). The result of CCCI is an inferred network of communication between cell types representing communication taking place at the time of sample collection.

One goal of many single-cell analyses is the inference of cellular changes between experimental contexts or treatment perturbations. In these cases, researchers may also seek to infer differences between cellular communication. However, most CCCI algorithms are designed to infer communication in a single sample rather than specifically compare changes in communication between measured systems. As hundreds to thousands of ligand-receptor signals between pairs of cell types in a data set, CCCI algorithms often rank ligand-receptor signals between cell types by a signaling score metric (Browaeys *et al.* 2020, Jin *et al.* 2021) or provide a list of ligand-receptor pairs contributing to signaling between cell types (Cherry *et al.* 2021). However, these measures of signaling often do not provide a measure of the signal’s variance among samples to quantify the significance of observed differences between conditions. Statistical methods are emerging to identify differential cell signaling based upon differential gene expression of ligands and receptors (Jin *et al.* 2021, Baruzzo *et al.* 2022, Browaeys *et al.* 2023, Lagger *et al.* 2023). There is a need for differential cell signaling methods that leverage biological replicates and capability for application to extended signaling cascades including ligands, receptors, and downstream response genes.

We developed Domino (Cherry *et al.* 2021) as a network-based CCCI algorithm that relates cell-cell communication from ligand-receptor signaling to the receptor activation of downstream transcription factors (TFs) in intracellular signaling. Previously, we demonstrated that the binary score of ligand-receptor interactions prioritized by Domino could be used to quantify differences in intercellular communication between cohorts of samples from different treatments tested in an immunotherapy clinical trial (Montagne *et al.* 2025). The sensitivity of this approach to sample size and cellular abundances was not assessed in these studies. Further extensions to compare conditions when limited sample numbers are pooled or to determine the changes to downstream transcription factor are also needed. To solve these problems, we develop the dominoSignal software as an R/Bioconductor package innovating upon Domino. This package has additional functionalities for comparing signaling networks between experimental conditions, inferring signals that utilize ligands and receptors that function as heteromeric complexes, and flexibly utilizing alternative ligand-receptor databases.

Several CCCI methods that utilize continuous scoring metrics of signaling have developed means for differential comparison of inferred signaling based on integrating statistical measures of gene differential expression (Browaeys *et al.* 2023) or derivation of a null distribution of scores through randomly assigning cells to groups being compared (Jin *et al.* 2021). As dominoSignal infers signaling as binary states of linkage between cell types via ligand and receptor pairs and linkage of receptors to transcription factors within a signaling cascade, we developed a statistical test for comparison of binarized signaling linkages called the differential cell signaling tests (DCST). The DCST utilizes Fisher’s Exact Test to statistically test the dependence of a signaling linkage differentially occurring between two groupings of samples represented in a scRNA-seq data set. Researchers can employ CCCI using dominoSignal in their analysis of scRNA-seq data formatted as a SingleCellExperiment (Amezquita *et al.* 2020) or Seurat (Satija *et al.* 2015) object given that they are familiar with the R statistical computing environment, can access a reference database of ligand-receptor gene pairs such as CellPhoneDB (Efremova *et al.* 2020), and can use a method of quantifying TF activity scores such as SCENIC (Van de Sande *et al.* 2020). We use the DCST to compare simulated signaling networks to assess the performance of identifying true differential signals, and we use DCST on real scRNA-seq data sets to demonstrate its functionality and the biological insights that can be gleaned from investigation of differential cell signaling using dominoSignal. We also compare the differential cell signaling linkages identified by dominoSignal to those identified by the comparable method scDiffCom (Lagger *et al.* 2023) and demonstrate the capability of extending the use of DCST to other methods of CCCI beyond dominoSignal by testing linkages inferred using the CellChat (Jin *et al.* 2021) method following binarization into on and off states.

## 2 Methods

### 2.1 dominoSignal for inference of inter- and intra-cellular signaling and DCST

dominoSignal is an R software package developed as an iteration upon the Domino R package developed by Cherry *et al.* (2021). Domino conducts CCCI in scRNA-seq data sets on the basis of inferring evidence of signal receipt based on Spearman correlation of genes encoding receptors with and a measure of TF activity in each cell. Ligand expression is then assessed based on the mean of scaled expression of the ligand-encoding genes by each cell type in the data set. Formatting and data structures are described in detail in Supplemental File 1 to store results. Our new software also creates signaling plots presented in the original version of Domino as published by Cherry *et al.* (2021) including TF feature expression by clusters, and heatmaps or networks of cumulative signaling between all clusters or specific linkages that each cluster takes part in. dominoSignal adds capabilities to render circos plots of the expression of ligands signaling to a receptor inferred to be active where the width of chords leaving cell type arcs corresponds to magnitude of ligand gene expression. The DCST uses Fisher’s Exact Test for statistics and returns the odds ratio of signaling taking place among subjects in the reference group relative to signaling taking place in subjects of the alternative group and the derived *P*-value are provided. Adjusted *P*-values (p.adj) are calculated using Benjamini-Hochberg false-discovery rate correction as implemented in the p.adjust function in the R stats package (v 4.2.0). This adjustment accounts for all unique linkages incoming to the cell type across all subjects. Circos plots were generated using circlize (v 0.4.15).

### 2.2 Parameters for dominoSignal cell-cell communication inference on real data sets

For all the real single-cell datasets, CCCI was conducted with dominoSignal (v1.0.6). All TF activity scoring was conducted using SCENIC (v 0.11.0) (Van de Sande *et al.* 2020). Ligand-receptor pairs for human gene expression data were obtained from CellPhoneDB (v4.0) (Efremova *et al.* 2020), and pairs for murine gene expression data were obtained from CellTalkDB (v1.0) (Shao *et al.* 2021). Unless otherwise specified, all instances of using dominoSignal were conducted using parameters min_tf_pval = 0.001, rec_tf_cor_threshold = 0.15, min_rec_percentage = 0.05, and no limits on the number of TFs inferred as active in a cell type or receptors linked to a transcription factor. Cell types were identified as ligand senders in intracellular linkages if the mean scaled expression of the ligand was greater than 0.

### 2.3 Parameters for additional CCCI methods

The PDAC reference data set was also subjected to CCCI using scDiffCom (v. 1.2.0) (Lagger *et al.* 2023). The run_interaction_analysis function was used on a Seurat object including all samples with LRI_specties set as “human,” seurat_celltype_id set as “crosstalk_celltype,” and seurat_condition_id set as “PDAC_class.” Interactions were considered statistically significant if they had a Benjamini-Hochberg adjusted *P*-value less than 0.05 and an absolute log-fold-change greater than 1. To run dominoSignal CCCI using the scDiffCom ligand-receptor reference database, the scDiffCom reference was obtained using the human_DB function in the scDiffCom package. Detected interactions were considered statistically significant if the Benjamini-Hochberg *P*-value (PH_P_VALUE_DE) was less than 0.05 and the absolute log-fold change was greater than 1.

CellChat (v 2.2.0) (Jin *et al.* 2021) was run on log-normalized RNA counts from cells in each sample independently using the CellChat R package. Inference used gene expression features identified as ‘overexpressed’ by the identifyOverExpressedGenes function and all parameters were set to package defaults. Interactions via ligands and receptors were formatted as a linkage summary of active interactions by considering all interactions with a probability greater than 0.1 as active intercellular linkages. The linkage summary underwent DCST using the test_differential_linkages function in dominoSignal.

## 3 Results

### 3.1 The linkage summary structure organizes inferred intercellular and intracellular signaling interactions to facilitate testing of differential signals

**Figure 1:**
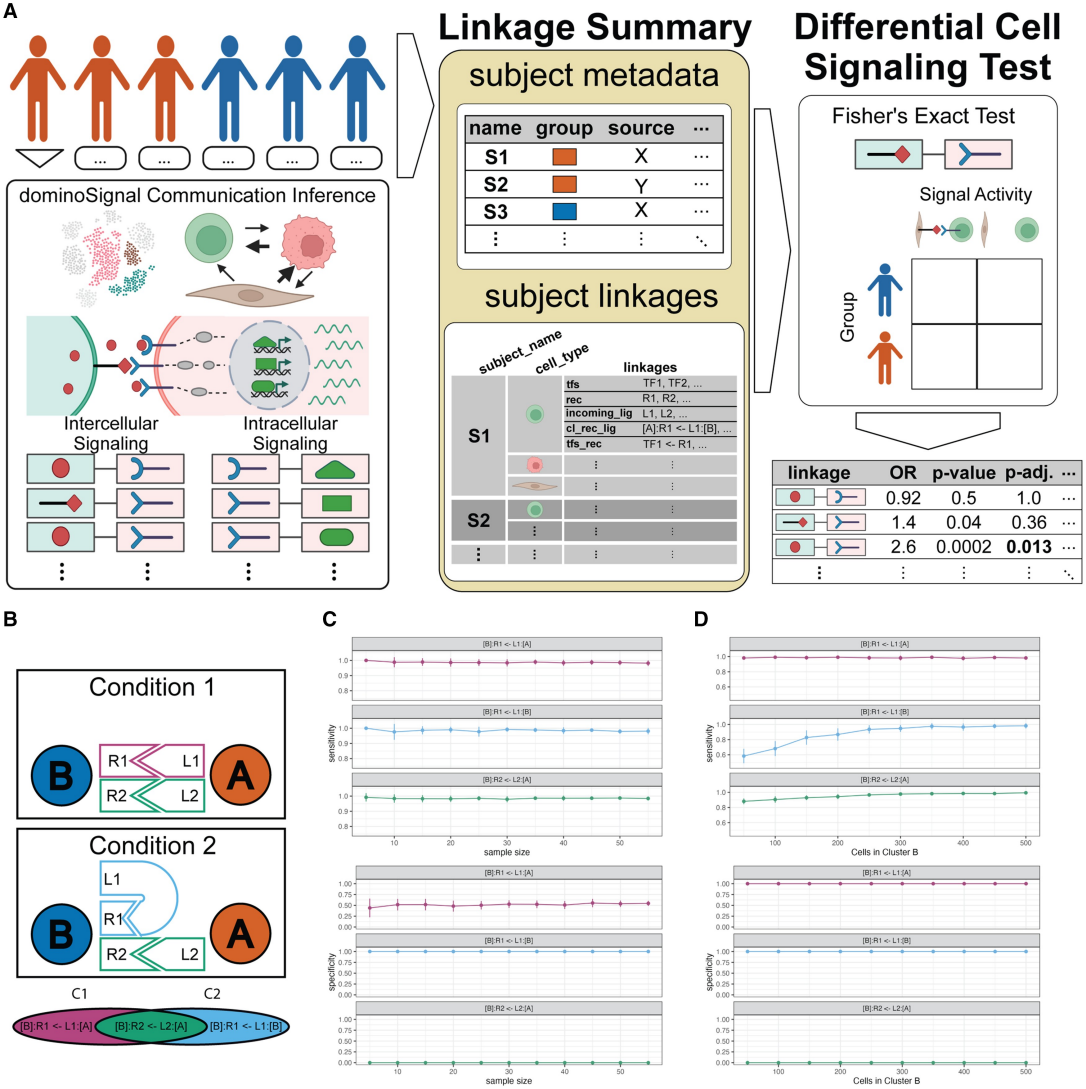
Framework of the differential cell signaling test (DCST). (A) For a single-cell RNA-seq data set comprised of cells from multiple subjects, the cells are divided into groups based on the subject they originated from. Cells from each subject undergo cell-cell communication inference (CCCI) with dominoSignal or other comparable CCCI methods, resulting in lists of inferred intercellular ligand-receptor interactions grouped by recipient cell type as well as inferred intracellular signals between receptors and transcription factors or signaling targets if the CCCI method permits. Inferred signals are organized in a data structure called a Linkage Summary consisting of a subject metadata table annotating the subjects considered and variables describing the subjects and subject linkages which is a nested list of subject names, the cell types present within the subject, and the inferred linkages from CCCI which may include active features (tfs, rec, incoming_lig) and signaling features (intercellular: cl_rec_lig; intracellular: tfs_rec). The differential signaling test of a given linkage counts the occurrences of this linkage among the subjects included based on their assignment to a grouping variable to be compared across and tests for dependence of the linkage being present based on the grouping variable using a Fisher’s Exact Test. For each linkage, the odds ratio of the linkage occurring based on the grouping variable and the derived *P*-value are provided as an output as well as the *P*-value with false-discovery rate adjustment for multiple tests of linkages involving the same recipient cell type. (B–D) Experiment on simulated single cell signaling network. (B) Graphical outline of simulated networks from two conditions, C1 and C2. Each condition has two cell type clusters, A and B, capable of interaction via ligand-receptor pairs L1 to R1 and L2 to R2. The Venn diagram shows interactions taking place specifically in C1 (magenta), C2 (blue), or are equally likely in both (green). (C) Sensitivity and specificity of detecting each interaction as significantly differential by DCST across simulations with different numbers of samples in the conditions being compared. (D) Sensitivity and specificity of DCST across different number of cells in cluster B. Dots are mean values across 10 unique initializations of the simulation parameters including standard deviations as vertical bars above and below the dots.

Briefly, Domino infers signaling by identifying TFs with differentially high activity in each cell type in a data set. The correlation between these TFs and receptor gene expression is assessed on a dataset-wide basis to establish TF-receptor linkages. An intracellular linkage between the receptor and TF is established if the TF is among those enriched in the cell type and a sufficient percentage of the cells in that cell type express the receptor. The presence of the intracellular linkage constitutes active receipt of signaling via the receptor in that cell type. Expression of the receptor’s ligands is then assessed by visualization of mean scaled expression of the ligands by cell types in the data set. dominoSignal, as an innovation upon Domino, provides discrete indications of signaling activity through the collation of linkages in which cell types participate. The DCST leverages this discrete quantification as the basis to compare inferred changes in cell-cell communication and is now implemented as a generalizable software for differential signaling analyses in scRNA-seq data sets measuring multiple experimental conditions for comparison (Fig. 1A).

To implement differential cell signaling analysis, we developed a pipeline in which we first perform CCCI for each sample in our scRNA-seq dataset independently. For example, if our dataset represents a cohort of tumors treated with different therapies, ligand-receptor network inference would first be performed for the data from each patient independently. This step ensures that inferred communication relies only on the multicellular environment for each sample. Changes in this cell-cell communication are then computed with a Fisher’s Exact Test designed to test the null hypothesis that the presence of an intercellular linkage between cell types is independent of a given comparative variable. In the example of multiple treatments described above, this analysis would employ Fisher’s Exact test on a contingency table of the number of subjects in each experimental group that include or lack the linkage being tested. Results are returned as a table including the odds ratio of the linkage being active in the reference group relative to the alternative group, the *P*-value derived from the Fisher’s Exact test, and FDR-adjusted *P*-values accounting for all interactions tested (Fig. 1A). This design based on the compilation of inferred linkages opens DCST to application with dominoSignal’s inferred networks or any CCCI method that infers signaling on a cell type basis given there are criteria for a linkage to be on or off. Thus, a statistical basis for differential signaling across experimental conditions can be used to prioritize signals. We developed a new Linkage Summary class described in detail in Supplemental File 1, which also allows for use of DCST with other CCCI methods that can be format inferred signaling as discrete linkages. This new infrastructure for differential cell signaling analysis is implemented in dominoSignal on Bioconductor.

We analyzed cell signaling changes using simulated data with a known ground truth to benchmark the performance of our differential signaling test under varying conditions. Briefly, we simulated single-cell data measuring two cell types (A or B) with set probabilities of ligand and receptor expression by each cell type that differ between two experimental conditions. We simulate simplified intercellular signaling with two simulation conditions (C1 and C2) where L1—R1 signaling from A to B ([B]: R1 <- L1: [A]) was more likely to occur in C1, L1—R1 autocrine signaling from B to B ([B]: R1 <- L1: [B]) was more likely to occur in C2, and L2—R2 signaling from A to B ([B]: R2 <- L2: [A]) was equally likely in both conditions (Fig. 1B). Varying the number of simulated samples in each condition being compared from *n* = 5 to *n* = 55, average specificity of identifying true differential linkages were consistently high, ranging from 1 (SD = 0) at sample size *n* = 5 to 0.978 (SD = 0.033) at sample size *n* = 25, but specificity ranged from 0.44 (SD = 0.22) at *n* = 5 to 0.55 (SD = 0.06) at *n* = 55, increasing with sample number (Fig. 1C, Supplemental Table S1). Varying the number of cells in cluster B in each condition, the sensitivity of detection of the autocrine signal in B cells as significantly differential increased with cell number from 0.616 (SD = 0.099) at 50 cells to 0.982 (SD = 0.035) at 500 cells, with specificity remaining consistently high across parameter changes (Fig. 1D, Supplemental Table S2). These simulations suggest that the accurate identification of differential cell signaling improves with the number of samples included and the number of cells representing the assessed cell types. We recommend that sample sizes of at least 15 and cell types with counts of at least 150 cells are sufficient for accurate identification of differential signals based on the proportions of initialized simulations where differential interactions were correctly identified (Supplemental File 2, Supplemental Table S3).

### 3.2 DCST analysis of a cohort of PDAC tumors demonstrates that classical PDAC exhibits increased fibroblast growth factor signaling compared to basal PDAC, validated in independent spatial transcriptomics data

**Figure 2:**
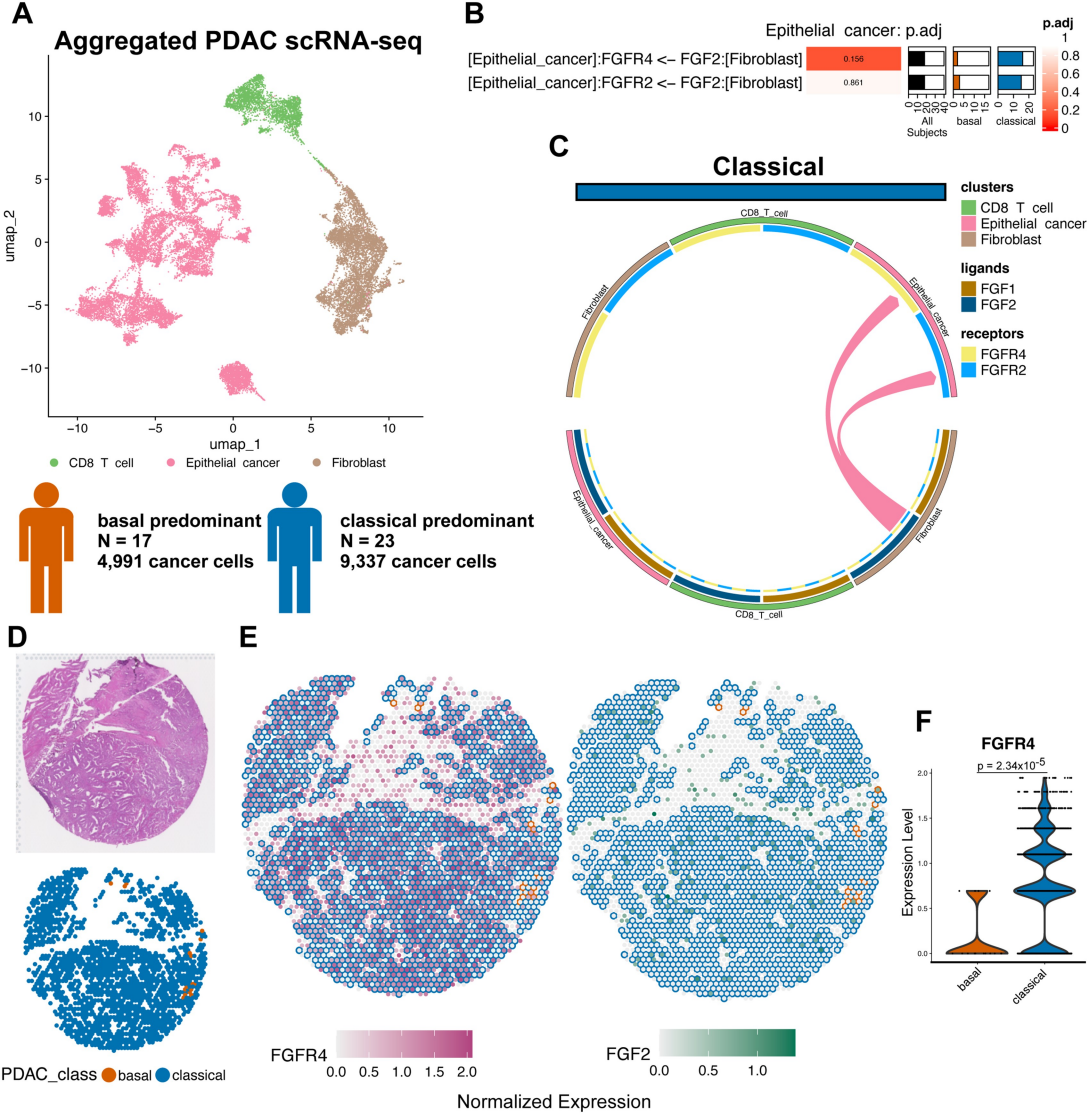
Consequences of DCST analysis comparing signaling between classical or basal PDAC subtypes. (A) UMAP of PDAC tumor cells scRNA-seq profiles from 40 subjects from Peng *et al*. (2019) and Steele *et al*. (2020) as annotated in Guinn *et al*. (2024) as CD8 T cell (green), epithelial cancer (pink), and fibroblast (brown). Among subjects’ epithelial cancer cells, 17 subjects had majority basal cells and 23 subjects had majority classical cells. (B) Leading differential signals from fibroblasts to epithelial cancer cells in classical or basal tumors. (C) Circos plot displaying differential signals via *FGF1* or *FGF2* to receptors *FGFR2* or *FGFR4* occurring in classical tumors. (D) Identification of classical (blue) and basal (orange) PDAC in Visium spatial transcriptomics data following the same module score approach employed on the scRNA-seq data. H&E image of PDAC tissue (left) is shown alongside Visium spots comprised of carcinoma cells colored by PDAC subtype (right). (E) Visualization of ligand and receptor expression in Visium spots where the spot center is colored by *FGFR4* (magenta, left) or *FGF2* (green, right) normalized expression and the border is colored based on the spot being classical PDAC (blue), basal PDAC (orange), or a non-carcinoma cell type (no border). (F) Violin plot of expression of *FGFR4* by basal PDAC (orange) and classical PDAC (blue) Visium spots. Overhead bar denotes the *P*-value from MAST test for differential gene expression.

We next sought to demonstrate the performance of our DCST methodology in dominoSignal to determine how transcriptional phenotypes can alter intercellular signaling. Transcriptomic studies of pancreatic ductal adenocarcinoma (PDAC) by Moffitt *et al.* (2015) described molecular subtypes of PDAC known as classical, characterized by increased cellular differentiation, and basal-like, characterized by greater plasticity and invasion potential. We expected that these differences in intrinsic properties of the neoplastic cells in a PDAC tumor would have ramifications on their interactions with non-neoplastic cells that constitute the PDAC microenvironment. To investigate this hypothesis, we obtained scRNA-seq data from the public data sets of Steele *et al.* (2020) and Peng *et al.* (2019), representing cells from 40 primary PDAC tumors. Cells were annotated according to Guinn *et al.* (2024). We focus our analysis on epithelial cancer cells, CD8+ T cells, and fibroblast cell types (Fig. 2A). Each tumor sample was classified into the subtypes defined by Moffitt *et al.* (2015) based on the proportion of epithelial tumor cells of each subtype, resulting in 24 classical tumors and 16 basal tumors (Supplemental File 3). Our Differential CCCI analysis was then applied based on these cellular and sample labels to demonstrate how this method could identify distinctions in basal and classical PDAC tumors in terms of how cancer cells interact with fibroblasts to shape the desmoplasia characteristic of PDAC and how CD8 T cells are hindered from cell killing by signaling within the PDAC tumor microenvironment.

Assessing differential signaling to epithelial cancer cells from fibroblasts identified 12 intercellular linkages differential between basal or classical subtypes before adjustment for multiple test correction (Supplemental File 4, Supplemental Table S4). The top linkage inferred with this analysis was increased signaling of *FGF2* (Fibroblast Growth Factor 2) from fibroblasts to *FGFR4* (Fibroblast Growth Factor Receptor 4) on epithelial cancer cells in classical PDAC as compared to basal (FDR-adjusted *P*-value 0.156, Fig. 2B and C). We further tested differential intracellular signaling from receptors to transcription factors between classical- and basal-predominant tumors. Assessing signaling involving *FGFR4*, signaling from *FGFR4* to *ETV4* (ETS Variant Transcription Factor 4) was more likely to occur in classical-predominant tumors (Supplemental Table S5). ETV4 is a transcription factor involved in cellular differentiation during development that has been previously associated with transduction of cell crowding mechano-sensation in human embryonic epithelium via FGFR endocytosis (Yang *et al.* 2024). To validate that *FGF2* signaling is a distinguishing feature of classical PDAC from basal PDAC, we assessed co-expression of *FGFR4* and *FGF2* in a spatial transcriptomics data set of PDAC from Bell *et al.* (2022) with co-occurrence of classical and basal PDAC cells within the same tumors. Among Visium (10X Genomics) spots annotated as cancer by Bell *et al.* spots were typed as classical or basal using the same module score approach as the PDAC scRNA-seq dataset (Fig. 2D). Cancer spots overlayed with *FGFR4* and *FGF2* gene expression showed a spatial absence of *FGFR4* expression in basal spots, demonstrating a diminished capacity to receive *FGF2* signals relative to classical PDAC (Fig. 2E). Expression of *FGFR4* was significantly lower in basal cells than classical [Fig. 2F, *P*-value = 2.34*10^−5^, MAST test (Finak *et al.* 2015)]. Whether this *FGF2* signaling difference represents a point of therapeutic intervention to drive carcinoma cells towards or away from a basal de-differentiated state warrants further investigation.

To determine the robustness of the identification of differential signaling interactions by DCST across methodologies, differential signaling was tested by scDiffCom (Lagger *et al.* 2023) and compared to results from DCST using dominoSignal CCCI. The scDiffCom analysis identified an enrichment of *FGF2* to *FGFR4* signaling from epithelial cancer cells to fibroblasts in classical-predominant tumors; however, the same signal was not detected in basal-predominant tumors because the expression of the *FGF2* ligand was below the limit of signaling detection (Supplemental Table S6). Thus, increased *FGF2* to *FGFR4* signaling in classical-predominant tumors was detected by both scDiffCom and DCST with dominoSignal relative to minimal detection in basal-predominant tumors. Among all signaling interactions detected from epithelial cancer cells to fibroblasts by scDiffCom, two ligand-receptor interactions from fibroblasts to epithelial cancer cells were found to be significantly enriched in classical-predominant tumors: *PRSS1* to *F2RL1* and *PRSS1* to *PARD3* (Supplemental Table S7). Each of these pairs was not annotated in the CellPhoneDB v4 ligand-receptor database used by dominoSignal in typical analysis pipelines. To ensure parity of signaling features being compared, DCST with dominoSignal was run using a ligand-receptor database including the 1,344 pairs from CellPhoneDB v4 and the 3,782 pairs present in the scDiffCom compendium of ligand-receptor pairs. Both differential interactions identified by scDiffCom were also identified in the extended dominoSignal DCST (Supplemental Table S8). Neither were statistically significant (FDR-adjusted *P*-value < 0.05) following multiple test correction as each interaction was only detected by dominoSignal in one of the 40 samples tested.

To demonstrate that DCST is applicable to additional CCCI methods beyond dominoSignal, we used CellChat (Jin *et al.* 2021) on each sample in the compendium of PDAC tumors and converted the results of signaling inference to a Linkage Summary by considering each inferred interaction as active if its probability of interaction was greater than 0.1. Using DCST to test for differential cell signaling between classical and basal tumors with Cell Chat CCCI, no interactions attained statistical significance (Supplemental Table S9). Thus, the DCST provides comparable results to contemporary methods for detecting differential signaling in scRNA-seq data and can be used with multiple CCCI methods including dominoSignal. However, differences in method of CCCI and the ligand-receptor feature set considered for CCCI will affect which differences in signaling are considered statistically significant.

### 3.3 Bootstrapped DCST analysis demonstrates that immune checkpoint inhibition halts immunosuppressive signaling from tumor-associated macrophages to exhausted CD8+ T cells in murine models of PDAC

Whereas the analyses above focused on leveraging our scRNA-seq data of immunotherapy combinations to benchmark algorithmic performance, previous studies demonstrated that the combination of PancVAX personalized vaccine with immune checkpoint inhibition is necessary to improve the survival of mice bearing Panc02 tumors (Kinkead *et al.* 2018). Mechanistic studies further demonstrated that this improvement in survival is attributable to reinvigoration of cytotoxic effector functions in CD8 T cells expressing exhaustion markers (Huff *et al.* 2023). Though the changes in gene expression by exhausted CD8 T cells between PancVAX and PancVAX + anti-PD-1 + anti-CTLA-4 have been assessed, intercellular signaling instigating the gene expression changes in exhausted CD8 T cells have yet to be identified. Thus, we sought to leverage our new DCST method and single-cell data to determine if the inclusion of anti-PD-1 and anti-CTLA-4 in a therapy regimen with PancVAX led to differential receipt of intercellular linkages by exhausted CD8 T cells as compared to PancVAX alone. The scRNA-seq dataset had limited sample sizes and pooled biological replicates for cost-effective hypothesis-generation scRNA-seq studies. To extend the applicability of the DCST to small data sets lacking sufficient biological replicates to apply the Fisher test to compare between groups of samples, we developed an approach to simulating biological replicates through bootstrapping of cells (Supplemental Files 3 and 5).

**Figure 3:**
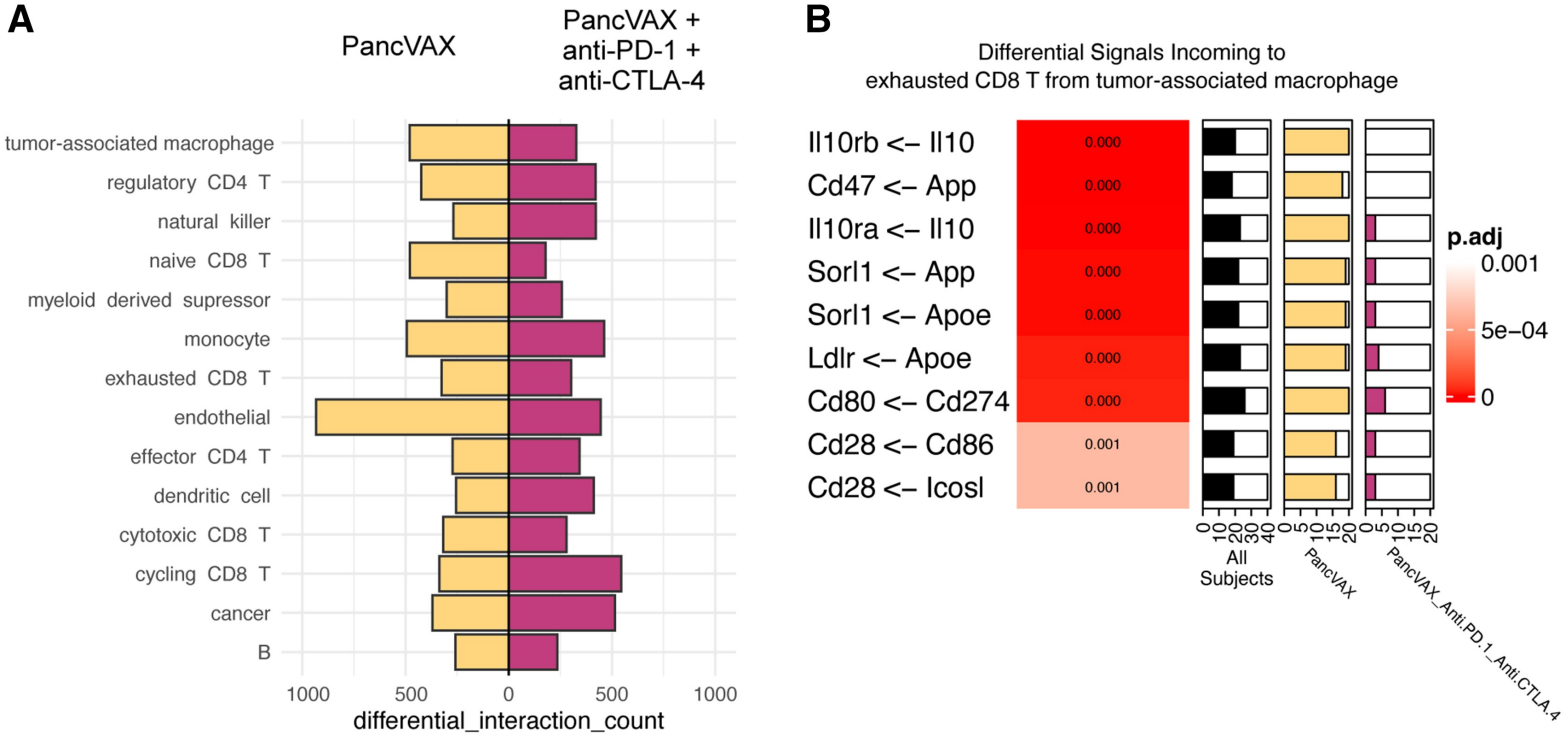
Immunosuppressive signaling by TAMs towards exhausted CD8 T cells is diminished in PancVAX + anti-PD-1 + anti-CTLA-4 treatment compared to PancVAX. (A) Counts of differential received signals in each cell type when comparing bootstrapped samples from PancVAX (*n* = 20, gold) to PancVAX + anti-PD-1 + anti-CTLA-4 (*n* = 20, magenta). (B) Top nine differential signals from tumor-associated macrophages to exhausted CD8 T cells with higher probability of occurring in PancVAX over PancVAX + anti-PD-1 + anti-CTLA-4. Signals are phrased in terms of “‘receptor’ <- ‘ligand’.” Gradient values correspond to FDR-adjusted *P*-value. Proportion bars display the number of bootstrapped subjects with active signaling in all subjects (black), PancVAX (gold), or PancVAX + anti-PD-1 + anti-CTLA-4 (magenta).

Bootstraps were generated from PancVAX and PancVAX + anti-PD-1 + anti-CTLA-4 cells and subjected to DCST of intercellular linkages for each recipient cell type (Fig. 3A, Supplemental Table S10). The top 9 differential intercellular linkages from tumor-associated macrophages to exhausted CD8 T cells, more likely to occur in PancVAX over PancVAX + anti-PD-1 + anti-CTLA-4 (Fig. 3B, Supplemental Table S10), revealed multiple immunosuppressive signals that diminished with addition of immune checkpoint inhibition. Among the differential signals sent by tumor-associated macrophages were interleukin-10 signaling (*Il10rb* <- *Il10*, *Il10ra* <- *Il10*), classically associated with suppressing cytotoxic T cell functions (Akdis and Blaser 2001), and apolipoprotein E signaling (*Sorl1* <- *Apoe*, *Ldlr* <- *Apoe*), demonstrated to be immunosuppressive in the context of pancreatic cancer (Kemp *et al.* 2021). *Cd80* <- *Cd274* signaling from macrophages, where *Cd274* encodes Programmed Death Ligand 1 (PD-L1), had greater occurrence in PancVAX over PancVAX + anti-PD-1 + anti-CTLA-4, an expected consequence of the anti-PD-1 therapy blocking PD-L1 to PD-1 signaling. Signaling to *Cd28* via ligands *Cd86* and *Icosl* was also more likely with PancVAX treatment. These signals are expected to be co-stimulatory to CD8 T cells (Hutloff *et al.* 1999, Esensten *et al.* 2016); however, treatment with PancVAX alone had worse survival than PancVAX + anti-PD-1 + anti-CTLA-4 (Huff *et al.* 2023), indicating that the differential suppressive signals overwhelm the stimulatory signals. In addition to the pairwise comparison of treatments, we ran a multilevel DCST comparing the untreated, PancVAX, and PancVAX + anti-PD-1 + anti-CTLA-4 treatments to identify intercellular signaling that varied across the 3 treatments. Among the most significant signals from tumor associated macrophages to exhausted CD8 T cells, suppressive *Il10* signaling occurred in all bootstraps except for the PancVAX + anti-PD-1 + anti-CTLA-4 treatment, and *Cd274* was absent from the untreated tumors, occurred in all bootstraps in PancVAX, and diminished again in PancVAX + anti-PD-1 + anti-CTLA-4 (Supplemental File 5, Supplemental Table S11). Use of DCST revealed signaling targets for therapeutic modulation towards enhancing anti-tumor immunity. Such insights are made possible by extension of this test to account for pooled samples.

### 3.4 DCST analysis of integrated scRNA-seq and scATAC-seq data improves linkage testing specificity to demonstrate that histone deacetylase inhibition alters inflammatory and immunosuppressive intratumoral signaling

An advantage of the dominoSignal CCCI method is its further association of receptor activation to downstream estimates of TF activation using regulon scores computed with SCENIC and the intracellular linkage of these scores with receptor expression. Single-cell Assay for Transposase Accessible Chromatin sequencing (scATAC-seq) data are known to refine estimates of transcription factor regulation, making it desirable to incorporate multi-omics data with both scRNA-seq and scATAC-seq in our DCST testing. To assess how the difference in TF activity quantification methodology would affect inferred signaling, we utilized scRNA-seq and scATAC-seq data reflecting mammary tumors collected from control (not treated) MMTV-PyMT transgenic mice (NT) and MMTV-PyMT mice treated with Entinostat (ENT), a selective class I histone deacetylase inhibitor, to rewire the immunosuppressive microenvironment of mammary tumors. Treatments were initiated when mice bearing early mammary adenomas were 80 days old and persisted for 15 days. Cells were annotated as cancer associated fibroblasts (CAF), lymphoid/Natural Killer (NK), myeloid and neoplastic based on marker gene expression or chromatin accessibility (Supplemental File 6).

**Figure 4:**
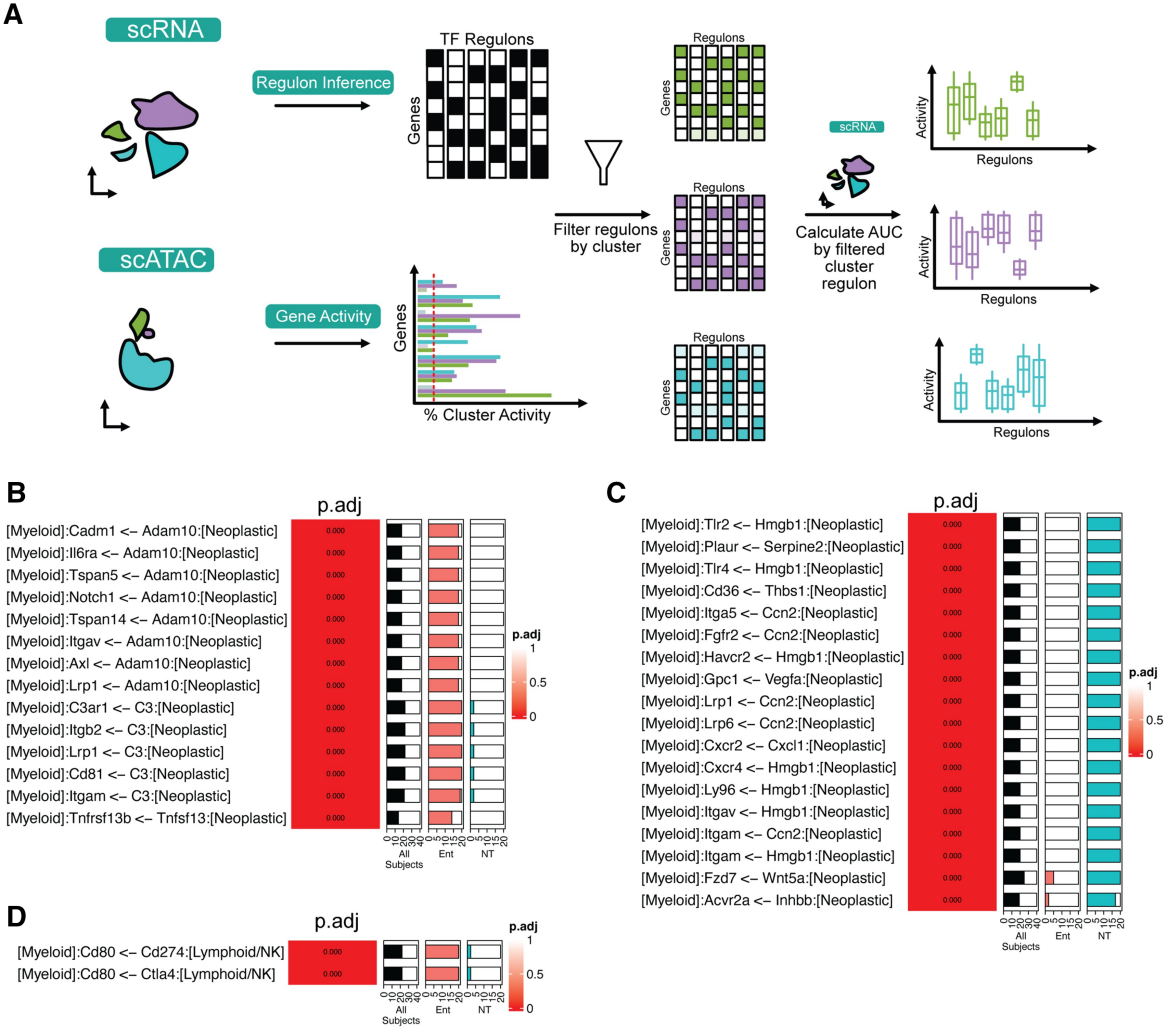
Targeted Regulon method integrates scATAC-seq accessibility data in dominoSignal inference to enhance profiling of inflammatory communication in Entinostat (Ent)-treated MMTV-PyMT transgenic mice. (A) Schematic illustrating novel approach to integrate scATAC-seq with scRNA-seq for dominoSignal analysis by refining SCENIC-inferred transcription factor regulons on a per-cell type basis using Signac gene activity estimates from scATAC-seq data with matching cell type labels. (B, C) Top differential signals from Neoplastic cells to Myeloid cells with higher probability of occurring in Ent (B) and no treatment (NT) (C) when comparing Ent and NT groups. (D) Top differential signals from Lymphoid/NK cells to Myeloid cells with higher probability of occurring in Ent when comparing Ent and NT treatment groups. Gradient values correspond to FDR-adjusted *P*-value. Proportion bars display the number of bootstrapped subjects with active signaling in all subjects (black), Ent (pink), or NT (blue).

In our dominoSignal analysis, SCENIC was selected for this estimation of TF activation as it refines the context-independent prior distribution of experimentally validated TF targets based upon scRNA-seq expression data. Therefore, we sought to expand this analysis to include estimates of TF activation from integrated scRNA-seq and scATAC-seq data. To leverage scATAC-seq data in improving SCENIC inference of TF activity from scRNA-seq datasets in our cohort, we developed Targeted Regulons, a novel approach to refining SCENIC-inferred TF regulons on a per-cell type basis using Signac gene activity estimates from scATAC-seq data with matching cell type labels (Fig. 4A). In brief, scRNA-seq data undergoes conventional SCENIC analysis up to the point of regulon inference. In scATAC-seq data collected from the same biological source as the scRNA-seq data, based on Signac gene activity scores, and genes are annotated as “accessible” in each cell type if 10% of the cells belonging to that type have non-zero activity for that gene. Cell type-specific regulons are then created by pruning the SCENIC-inferred regulons to remove target genes that are inaccessible within the cell type. These pruned regulons are then used as gene sets by AUCell to quantify TF activity in each scRNA-seq cell. These TF-activity values thus only incorporate a target gene in the estimate of TF activity if accessible for transcription and can natively be incorporated in our dominoSignal method. SCENIC+ (Bravo González-Blas *et al.* 2023) can also be used for this integration, but in our dataset we found that the meta-cell approach resulted in significant variation in estimates of TF scores due to substantial variation in the characteristics of generated meta-cells across different initializations in distinct software versions of SCENIC+ (Supplemental File 7) motivating our use of Targeted Regulons for downstream analysis in this study.

To assess the impact of integrating scATAC-seq data, we inferred from scRNA-seq data alone using SCENIC or paired scRNA-seq and scATAC-seq data using the new Targeted Regulons method in our MMTV-PyMT dataset. DCST was used to compare intracellular signaling inferred with dominoSignal using the Targeted Regulons method for quantifying TF activity as compared to SCENIC using transcription data alone. Comparing 20 bootstraps from NT cells using each methodology, inferred intracellular linkages were classified as being more likely to be identified with Targeted Regulons, with SCENIC, or equally likely with either method. In the Myeloid and Lymphoid/NK clusters, there were fewer intracellular linkages more likely to be inferred with Targeted Regulons than with SCENIC (Supplemental File 8). Conversely, Targeted Regulons inferred more intracellular signaling in Neoplastic and CAF cells (Supplemental File 8). In all cases, most regulons inferred from the method were shared between the Targeted Regulon and SCENIC analysis. This suggests the refinement of regulons to consider only accessible target genes hones inferred intracellular signaling in a cell-type dependent manner, likely varying based on the number of cells measured and heterogeneity in their cell subtypes.

To investigate the effects of ENT treatment on altering the communication networks in the MMTV-PyMT tumor microenvironment, intercellular signals received by each cell type were compared between sets of 20 bootstraps from the NT and ENT treatment groups using DCST on dominoSignal-inferred signaling. Among signals more likely to be received by myeloid cells from neoplastic cells in ENT compared to NT, ENT leads to increased receipt of *Adam10* metalloprotease signaling, increased complement 3 (*C3*) inflammatory signals, and inflammatory tumor necrosis factor signaling through *Tnfsf13* (Fig. 4B). In contrast, myeloid cells in NT tumors received more *Ccn2* (cellular communication network factor 2) signaling regulating ECM production (Rodrigues-Díez *et al.* 2022), more damage-associated molecular patterns via *Hmgb1*, and more growth signals via *Wnt5a* and *Vegfa* (Fig. 4C). Collectively, these findings indicate that while ENT treatment promotes features of a proinflammatory tumor microenvironment, concurrent increases in immune checkpoint signaling may offset these effects and account for the limited efficacy of ENT monotherapy in this model. Myeloid cells within ENT-treated tumors exhibited increased receipt of *Ctla4*-mediated signals from interacting lymphoid and NK cells via *Cd80* engagement (Fig. 4D). *Ctla4* is a central immune checkpoint receptor that suppresses cytotoxic T cell activation by antagonizing *Cd80*-dependent co-stimulatory signaling provided by antigen-presenting cells (Wei *et al.* 2017, Rowshanravan *et al.* 2018). In parallel, myeloid cells also received increased signaling from the lymphoid/NK compartment via *Cd274* (PD-L1)-*Cd80* interactions. Beyond its canonical role as a ligand for PD-1, *Cd274* can bind *Cd80* in cis or trans to inhibit CD28-mediated co-stimulation, thereby reinforcing immune suppression independently of PD-1 signaling (Butte *et al.* 2007, Sugiura *et al.* 2019, Zhao *et al.* 2020). Collectively, these signaling patterns indicate that while ENT treatment may enhance inflammatory cues and immune cell engagement within the tumor microenvironment, concurrent activation of *Ctla4*- and *Cd274*-*Cd80*-mediated inhibitory pathways may functionally restrain effective antitumor immunity and limit immune-mediated tumor repression.

## 4 Discussion

We present the DCST as a novel approach to testing differential signaling networks of inferred cell-cell communication in scRNA-seq data. We implement DCST using our CCCI method Domino as a foundation and develop a new R/Bioconductor package dominoSignal for Domino and our DCST methods. dominoSignal includes a Linkage Summary data format for efficient storage of signaling inferred as intercellular and intracellular linkages based on recipient cell type from many samples. The DCST provides a statistical basis for the comparison of these inferred signals within and between cell types across discrete independent variables in scRNA-seq data sets with many biological variables. We also include a bootstrapping approach for applications of DCST to data sets without annotation of biological variables to expand the contexts to which researchers can apply DCST.

We demonstrate through both simulated and real-world examples that the DCST method in dominoSignal distinguishes complex inter- and intra-cellular signaling changes between biological phenotypes and treatment conditions. Simulated data experiments identify sample sizes sufficient for accurate calling of differential cell signaling, and our bootstrapping approach provide a robust approach to assessing differential cell signaling in experiments with few replicates with the caveat of inconsistent results for cell types represented by too few cells. Our applications to real-world data show how the assessment of differential cell signaling can guide understanding of signaling interactions associated with distinct molecular states of pancreatic cancer, provide mechanistic insight into consequences of combination immunotherapy on networks of cellular interactions within tumors, and disentangle consequences of altered signaling via receptors with context-dependent signaling outcomes. We also use DCST to compare the methodologic basis of signaling networks inferred by dominoSignal when using SCENIC, RNA-seq-based, TF activity inference to dominoSignal using integrated scATAC-seq TF inference through the Targeted Regulons method. The cell-type dependence of the impact of scATAC-seq data on refining the number of target TFs reflects the importance of future work more fully evaluating the impact of scATAC-seq on inference of cell-cell communication in a cell-type dependent manner. DCST leverages dominoSignal’s quantification of signaling in each sample as intercellular and intracellular linkages in binary on/off states to test dependence of these signals occurring upon a discrete sample variable. The Linkage Summary format of storing incoming signals to cell types is agnostic of the method of inference. It could be employed to compare networks inferred by other CCCI methods in addition to dominoSignal, as demonstrated by using binarization of signaling inference from CellChat. However, future work is needed to evaluate the sensitivity of the differential ligand-receptor analysis to the threshold used for binarization or extend DCST to compare continuous scores.

We acknowledge that DCST is not appropriate for all comparisons of inferred signaling. DCST’s basis in Fisher’s Exact Test is well suited for comparisons of pairs of fixed states or groupings of samples. This test cannot estimate effect sizes of incremental changes in continuous variables that describe samples. This could be important to investigations such as signaling consequences of increasing drug concentration or assumed additive effects of sample genotype. The binarization of signaling in DCST facilitates its application with dominoSignal and other CCCI methods, but it does preclude interpretation of signaling intensity aside from the consistency of signaling taking place among biological replicates. We also identified large sample sizes and cell counts within cell types as critical prerequisites to accurate identification of differential signals. Large sample sizes are often difficult to attain in scRNA-seq experiments due to financial cost (Svensson *et al.* 2018). Tests must also consider hundreds to thousands of incoming linkages to a cell type, necessitating multiple-test correction of the derived *P*-values. We demonstrate that increasing the number of unique ligand-receptor pairs tested by appending the scDiffCom ligand-receptor reference to CellPhoneDB does diminish ability to detect significantly different signaling due to increasing adjustment for multiple test correction. These considerations of sample size and cell number for increased statistical power are best addressed by using compendiums of multiple data sets, as demonstrated in the PDAC single-cell RNA-seq datasets used in this study (Guinn *et al.* 2024), or large single-cell atlases representative of specific tissues or disease states. Further innovations upon the DCST method may include grouping ligand-receptor pairs into groups of functionally related signals to decrease the number of independent tests conducted and to account for receptor families whose activation trigger shared gene programs.

For scRNA-seq experiments with small sample sizes or pooled samples, we provide a bootstrapping approach that uses observed data to estimate variation among biological replicates and demonstrated that this bootstrapping approach does not result in spurious identification of differential signals given that sufficiently large sample sizes are reached. This allows for the assessment of differential cell signaling in small-scale experiments. However, we acknowledge that bootstrapping results will better represent gene expression of the most ubiquitously expressed ligands and receptors while missing signals driven by rare cell populations within cell types. dominoSignal identifies cell types as ligand senders based on positive mean scaled expression of the ligand among cells of that type, which may also diminish the method’s capability to identify sparse, transiently expressed ligands in an experiment. Raising or lowering the expression threshold for ligand senders is a means by which researchers can make the inference of signaling more or less strict with dominoSignal, but methods to better account for the transcription dynamics of ligand-encoding genes is a direction of ongoing investigation.

DCST has the potential for wide application to compare signaling networks inferred in diverse experimental settings. Future directions of research may include the use of alternative statistical bases for comparing signaling interactions among samples. One such approach may be the use of logistic regression basis for DCST. The binary representation of an inferred signal’s activation state is well suited to dichotomous outcomes assessed by logistic regression. The generalized linear model basis of logistic regression allows for the consideration of covariate interaction effects on the signaling outcome (Hosmer and Lemeshow 2000). However, even larger sample sizes are expected to be necessary to power usage of logistic regression compared to small sample sizes of the non-parametric Fisher’s Exact Test where cells in the contingency table may sometimes be less than 5. In addition to alternative statistical tests, criteria for the formatting of CCCI results derived from other methods should be explored to enable applications of DCST to additional CCCI methods beyond dominoSignal and CellChat as well as direct comparisons of the signals identified by different CCCI methods on the same data set.

## Supplementary Material

btag089_Supplementary_Data

## Data Availability

This study analyzes single-cell RNA-seq data from human PDAC tumors obtained from the datasets compiled and annotated as described in Guinn *et al.* (2024) and Visium spatial transcriptomics data as described in Bell *et al.* (2022) and Lyman *et al.* (2025), using data in NIH Gene Expression Omnibus (GEO) repositories GSE254829 and GSE294669. Single-cell RNA-seq data of vaccine and immune checkpoint inhibitor treatment of a subcutaneous, murine model of PDAC generated from the Panc02 cell line are obtained from Huff *et al.* (2023), and use the cellular annotations from that study (GEO GSE244992). Finally, new data paired single-cell RNA-seq and ATAC-seq data from untreated and Entinostat treated MMTV-PyMT mice are also used for analysis. Detailed methods for the processing of these datasets are described in Supplemental File 3. The dominoSignal package is available through Bioconductor (https://www.bioconductor.org/packages/release/bioc/html/dominoSignal.html). Scripts used for analysis presented in this manuscript are available from Zenodo (doi: 10.5281/zenodo.18329130) and https://github.com/FertigLab/Differential_Cell_Signaling_Test.
