# Supplementary material for: Differential cell signaling testing for cell-cell communication inference from single-cell data by dominoSignal
Source: Bioinformatics. 2026 Feb 26;42(3):btag089. doi: 10.1093/bioinformatics/btag089 (PMC12998610; doi:10.1093/bioinformatics/btag089)
Supplement: btag089_Supplementary_Data [file btag089_supplementary_data.zip › Supplemental File 7.docx]

**Supplemental File 7: SCENIC+ transcription factor scoring of unmatched scRNA-seq and scATAC-seq data varies between software versions and is sensitive to meta-cell selection**

As one means to integrate scRNA-seq and scATAC-seq data collected from MMTV-PyMT tumors to infer transcription factor activation, we leveraged SCENIC+ (Bravo González-Blas *et al.* 2023). The goal was to use transcription factor (TF) activity scoring from SCENIC+ in dominoSignal’s inference of signal receipt by correlation of TF scores with receptor gene expression (Cherry *et al.* 2021). In one analysis, we used SCENIC+ version v1.0 (scp01) and in the second updated version v1.0.a1 (scp02). Comparing the results of dominoSignal inference using the different SCENIC+ results as TF activity scores, we found significant deviation between results generated with the two different versions of software.

In a direct comparison of the inferred TF activity scores from scp01 and scp02, mean activity scores among meta cells in each cell type were not significantly correlated in CAF and Neoplastic cell types (Supplemental File 7 Figure 1). Mean TF activities were correlated in Myeloid and Lymphoid/NK cell types but have a less than perfect correlation that would reflect perfect reproducibility.

To assess the propagation of differences in TF activity scoring into dominoSignal signaling inference, we assessed the Spearman correlation between Macrophage Inhibitory Factor (Mif) receptor genes *Ackr3*, *Cd44*, *Cd74*, *Cxcr2*, and *Cxcr4* and TFs that were linked to these receptors in the original scp01: *Bnc2*, *Elf1*, *Etv6*, *Ikzf3*, and *Mitf*. dominoSignal’s basis for intracellular linkage between TFs and receptors is Spearman correlation (rho) of TF activity with receptor gene expression above a threshold value, defaulting to a rho = 0.15 threshold. Of the 14 intercellular linkages identified in the ENT treatment between these TFs and receptors in scp01, only 7 of the same linkages were formed in scp02, and 2 new linkages were inferred in scp02 that had not been identified in scp01. We note this variation can either arise from updates to the algorithm underlying SCENIC+ or the random selection of meta-cells used to compute TF activity in our unpaired scRNA-seq and scATAC-seq design.


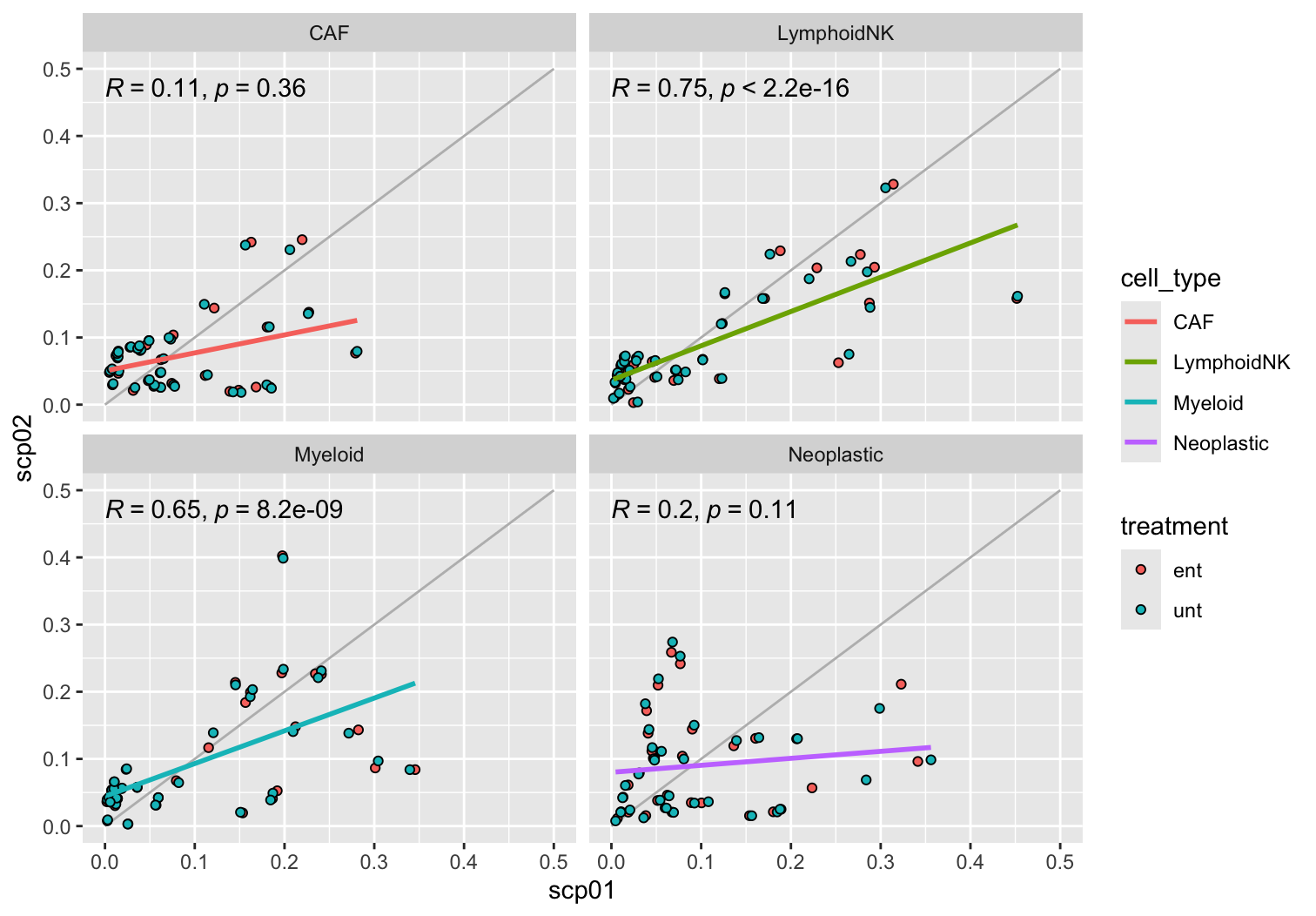


**Figure 1: Mean cell type SCENIC+ TF scores are not correlated between version 1.0 and version 1.0.a1 analysis results.** Scatterplots of mean TF scores inferred by SCENIC+ in CAF (top-left), Lymphoid/NK (top-right), Myeloid (bottom-left), and Neoplasitc (bottom-right) cell types under software versions v1.0 (scp01) or version v1.0.a1 (scp02). Each point represents a TF quantified using both versions of SCENIC+ in the entinostat (ENT)-treated cells (pink) or the non-treated (NT) cells (blue). Points are overlayed with a line representing linear regression of scp02 scores over scp01 scores considering all TFs from both treatments. Panels are overlayed with correlation test results including the pearson correlation coefficient (R) and the probability of rejecting the null hypothesis that the correlation between scores is 0 (p). The grey line represents a hypothetical perfect correlation (R = 1).


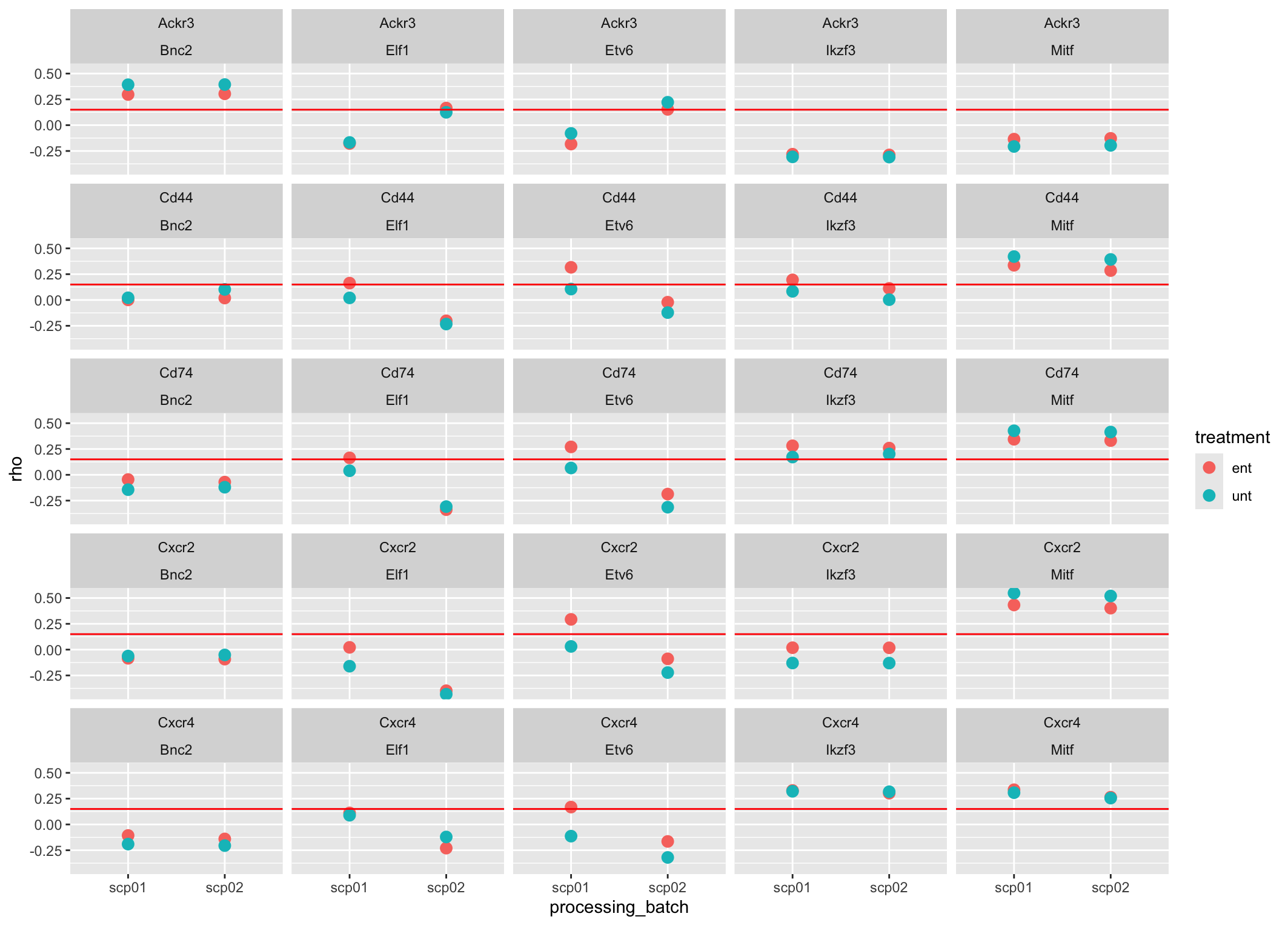


**Figure 2: Correlations between receptors of Mif and transcription factors linked by dominoSignal in SCENIC+ version 1.0 result do not attain correlation for linkage in version 1.0.a1**. Dot plots showing the Spearman correlation coefficients (rho) between receptors of Macrophage Inhibitory Factor (*Mif*) ligand and transcription factors among SCENIC+ meta cells from entinostat (ENT)-treated cells (pink) or the non-treated (NT) cells (blue) under version v1.0 (scp01) or version v1.0.a1 (scp02). Panels are annotated by the receptor (top label) and TF (bottom label) tested for correlation. The red horizontal line denotes the threshold of Spearman correlation sufficient to establish a linkage between receptor and TF.

Works Cited

Bravo González-Blas C, De Winter S, Hulselmans G *et al.* SCENIC+: single-cell multiomic inference of enhancers and gene regulatory networks. *Nat Methods* 2023;**20**:1355–67.

Cherry C, Maestas DR, Han J *et al.* Computational reconstruction of the signalling networks surrounding implanted biomaterials from single-cell transcriptomics. *Nat Biomed Eng* 2021;**5**:1228–38.
