# Supplementary material for: Differential cell signaling testing for cell-cell communication inference from single-cell data by dominoSignal
Source: Bioinformatics. 2026 Feb 26;42(3):btag089. doi: 10.1093/bioinformatics/btag089 (PMC12998610; doi:10.1093/bioinformatics/btag089)
Supplement: btag089_Supplementary_Data [file btag089_supplementary_data.zip › Supplemental File 8.docx]

**Supplemental File 8: Comparison of SCENIC and Targeted Regulons as the transcription factor quantification basis for dominoSignal inference**

DCST is capable of applications in comparing methodological bases for cell-cell communication inference in addition to assessing differences in signaling due to experimental conditions. Using bootstraps from the non-treated (NT) tumors from MMTV-PyMT mice, we compared the intracellular signals inferred when using transcription factor (TF) scoring based on Targeted Regulons that integrate scRNA-seq and scATAC-seq data or SCENIC scores that use scRNA-seq data alone. Intracellular signals between receptors and TFs from each methodologic basis were compared using DCST on each cell type and categorized as being significantly more likely to be inferred using Targeted Regulons or SCENIC, based on having an FDR-adjusted p-value less than 0.05. Intracellular signals equally likely to be found by both methods were categorized as insignificant.

The pruning of putative SCENIC regulons by the Targeted Regulons method based on chromatin accessibility of target genes was expected to decrease the number of intracellular signals inferred by removing false-positive TF-target associations. We found that this decreased number of intracellular signaling inference only occurred on a cell type-dependent basis. The immune cell clusters exhibited decreased numbers of intracellular linkages using Targeted Regulons compared to SCENIC, with myeloid cells having 791 fewer exclusive linkages and Lymphoid/NK having 531 fewer. However, CAFs and Neoplastic cells had more inferred linkages using Targeted Regulons with 296 more linkages and 1097 more linkages, respectively. These results show the dominoSignal inference may be refined by using a TF-scoring methodology that integrates chromatin accessibility data, but the improvement is not uniform across all cell types.


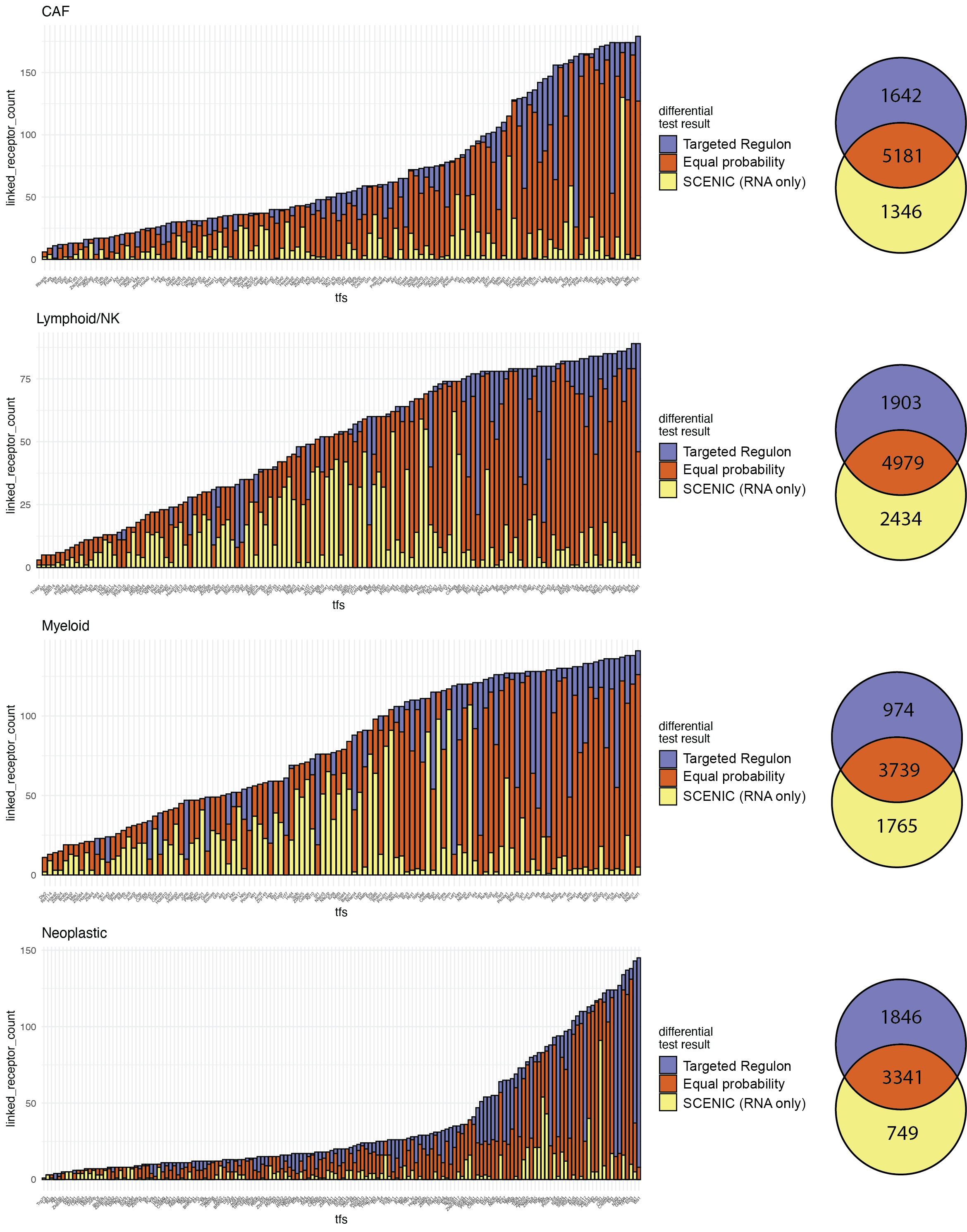


**Figure 1: Counting intracellular signals differentially inferred using dominoSignal with Targeted Regulons or SCENIC**. Stacked bar plots for each transcription factor (TF) in common between the scoring methods count the number of unique receptors found linked to the TFs across the generated bootstraps. Segments of the bar plots are colored based on whether the TF-receptor linkage was more likely to inferred using Targeted Regulons (violet), SCENIC (yellow), or equally likely by both methods (orange). The numbers of unique linkages across all TFs in the cell type are collated as a Venn diagram to the right.
