# Supplementary material for: Differential cell signaling testing for cell-cell communication inference from single-cell data by dominoSignal
Source: Bioinformatics. 2026 Feb 26;42(3):btag089. doi: 10.1093/bioinformatics/btag089 (PMC12998610; doi:10.1093/bioinformatics/btag089)
Supplement: btag089_Supplementary_Data [file btag089_supplementary_data.zip › Supplemental_Table_Legends.docx]

**Supplemental Tables**

Supplemental Table 1: Sensitivity and specificity of DCST on simulated data with varying sample sizes

Supplemental Table 2: Sensitivity and specificity of DCST on simulated data with varying cell number in recipient cell type

Supplemental Table 3: Proportion of DCST simulations with varying sample number where differential intercellular signaling was detected

Supplemental Table 4: dominoSignal DCST results comparing intercellular linkages incoming to epithelial cancer cells between basal and classical PDAC samples

Supplemental Table 5: dominoSignal DCST results comparing intracellular linkages within epithelial cancer cells between basal and classical PDAC samples

Supplemental Table 6: Raw scDiffCom results of inferred intercellular signaling by FGF2 to FGFR4 comparing basal and classical PDAC samples

Supplemental Table 7: Significantly different intercellular signaling inferred by scDiffCom comparing basal and classical PDAC samples

Supplemental Table 8: dominoSignal DCST results comparing intercellular linkages incoming to epithelial cancer cells between basal and classical PDAC samples considering ligand-receptor pairs from both CellPhoneDB and scDiffCom

Supplemental Table 9: CellChat DCST results comparing intercellular linkages incoming to epithelial cancer cells between basal and classical PDAC samples

Supplemental Table 10: dominoSignal DCST results comparing intercellular linkages incoming to exhausted CD8 T cells between PancVAX and PancVAX + anti-PD-1 + anti-CTLA-4 bootstraps from Panc02 tumors

Supplemental Table 11: Multilevel dominoSignal DCST results comparing intercellular linkages incoming to exhausted CD8 T cells between Untreated, PancVAX, and PancVAX + anti-PD-1 + anti-CTLA-4 bootstraps from Panc02 tumors
