# Supplementary material for: Differential cell signaling testing for cell-cell communication inference from single-cell data by dominoSignal
Source: Bioinformatics. 2026 Feb 26;42(3):btag089. doi: 10.1093/bioinformatics/btag089 (PMC12998610; doi:10.1093/bioinformatics/btag089)
Supplement: btag089_Supplementary_Data [file btag089_supplementary_data.zip › Supplemental File 1.docx]

**Supplemental File 1: Details about the dominoSignal functions and software vignettes and usage of dominoSignal**

Vignettes tutorizing the usage of dominoSignal, plotting dominoSignal results, navigating domino objects, and using SCENIC for TF activity quantification is available from <https://fertiglab.github.io/dominoSignal/index.html> using a reference scRNA-seq data set of 4000 peripheral blood mononuclear cells collected by 10X Genomics (10X Genomics).

**The linkage summary structure organizes inferred intercellular and intracellular signaling interactions to facilitate testing of differential signals**

Signaling taking place between cell types via ligands and receptors is compiled in our software as **intercellular linkages**. In dominoSignal, these linkages are named following the format of:

[‘recipient cell type’]:‘receptor’ <- ‘ligand’:[‘sender cell type’]

Linkage naming initiates with the recipient cell because dominoSignal’s inference begins with evidence of receptor expression correlated with transcription factor activity in the recipient cell type. Network-based methods for CCCI, including Domino (Cherry *et al.*, 2021) and NicheNet (Browaeys *et al.*, 2020), further extend the network inference to estimate activation of TFs or individual target genes downstream of each receptor (Su *et al.*, 2024). To account for inferred signaling between receptors and TFs or target genes, our software is also designed to compile these interactions as **intracellular linkages**.

Interactions are encoded in dominoSignal by storing nested lists within a Linkage Summary object in a nesting hierarchy of subject identifier > receiver cell type > linkage type (Figure 1). Linkages are organized based on receiving cell type, following the recipient to sender direction of dominoSignal’s CCCI approach. The linkage summary object also stores a table of meta data annotating features of each subject across which their signaling interactions can be compared to facilitate the implementation of the Fisher’s Exact Test for differential communication. The Linkage Summary more efficiently uses computer memory compared to maintaining Domino data objects for each of the samples as these objects also contain large matrices of gene expression data, calculated correlation coefficients, and ligand-receptor pairing references.

**Ligand-receptor and downstream transcription factor mapping**

dominoSignal CCCI on a scRNA-seq data set requires the data set’s raw RNA count matrix, the normalized and scaled RNA matrix, a TF by cell TF activity score matrix, and a table describing the possible ligand-receptor pairs called an rl_map. Optionally, researchers may include a character vector of cell assignments to clusters or cell types so that signal-receipt on a per-cell type basis may be assessed and a list of genes inferred to be regulatory targets of each TF as a “regulon” which can be used in the dominoSignal analysis to remove TF-receptor linkages that may be attributable to TF activation driving receptor expression. The basis of TF activity quantification is up to the discretion of the user. Conventionally, Domino and dominoSignal have used SCENIC (Van de Sande *et al.*, 2020) as the basis of TF-activity quantification, but any method that results in a TF by cell matrix of activity scores is a valid input. The “rl_map” must include columns annotating the interacting ligand-receptor pairs. These include: int_pair, the names of the interacting ligand and receptor separated by “ & ”; gene_A, the gene or genes encoding partner A; gene_B, the gene or genes encoding partner B; type_A, (“L”, “R”) - indicates whether partner A is a ligand (“L”) or receptor (“R”); and type_B, (“L”, “R”) - indicates whether partner B is a ligand (“L”) or receptor (“R”). For ligands or receptors that function as heteromeric complexes encoded by multiple genes, the names of all genes in the complex are included in the gene_A or gene_B columns separated by commas. dominoSignal includes a helper function, *create_rl_map_cellphonedb*, to format database tables from CellPhoneDB (Efremova *et al.*, 2020) annotating human ligand-receptor pairs into an rl_map format. rl_maps for mouse ligand-receptor interactions were generated from CellTalkDB (Shao *et al.*, 2021).

The first stage of the dominoSignal analysis is carried out by the *create_domino* function. This function takes the required data described above as inputs with additional parameters “use_clusters” which dictates if cell type assignments will be considered for receptor activation and ligand expression as well as “use_complexes” which dictates if heteromeric complex ligands and receptors included in the rl_map will be considered during CCCI. The result of *create_*domino is a domino object, an S4 class that stores the input data used for CCCI as well as information on the tests for signaling inference and the resulting signaling network. *create_domino* begins CCCI by testing for enrichment of TF activity in each cell type by conducting a one-sided Wilcoxon rank-sum test of whether each TF’s mean score in the cell type is significantly greater than the mean score in all other cells. A matrix of p-values from the Wilcoxon rank-sum tests are stored in the “de” slot of the resulting domino object. Spearman correlations between the TF activities and receptor gene expression are then assessed across all cells in the data set. Calculated Spearman correlation coefficients are stored in the “cor” slot of the domino object. For complex receptors encoded by multiple genes, the stored correlation values are the median coefficients among all component genes. If a list of TF regulons is provided to the “tf_targets” argument to *create_domino*, receptor that is part of a TF’s regulon will be set to 0 correlation to avoid assigning linkage between a TF that drives a receptor’s expression rather than the receptor driving TF activity. With TF enrichment and receptor-TF correlations calculated and stored in the domino object, the next step pertains to setting parameters for assigning intracellular and intercellular linkages.

The next step of dominoSignal analysis is carried out by the *build_domino* function. This function takes the domino object resulting from create_domino as an argument as well as parameters for assigning linkages. “min_tf_pval” sets the maximum Wilcoxon rank-sum test p-value for assigning a TF as enriched in each cell type. A cell type’s enriched TFs are stored as a list within the domino object’s “linkages$clust_tf” slot. “rec_tf_cor_threshold” sets the minimum Spearman correlation coefficient between a TF and receptor to assign an intracellular linkage between them. Data set-wide TF-rec linkages are stored in the “linkages$tf_rec” slot. Intracellular linkages within cell types are assigned on the basis of enrichment of the TF in the cell type as well as non-zero expression of the receptor in a minimum percentage of cells within the cell type specified by the “min_rec_percentage” argument to *build_domino*. Active receptors for each cell type are stored in the “linkages$clust_rec” slot and their intracellular linkages with TFs is stored within “linkages$clust_tf_rec”. Intercellular linkages annotating a sender cell type, ligand, receptor, and receiving cell type can be annotated in “linkages$rec_lig_cl” based on mean scaled ligands expression above a threshold using the *add_intercellular_linkages* function where the threshold is specified by the “signal_threshold” parameters.

**The Linkage Summary class structure**

The Linkage Summary is an S4 class object in the R computing environment defined within the dominoSignal R package to organize inferred cell-cell communication via ligands to receptors and intracellular associations of receptors with gene or TF targets of activation. The Linkage Summary object stores the linkages slot from multiple domino objects to facilitate their comparison and save space in computer memory by not including the other data in the domino objects such as expression matrices. The linkage summary consists of three slots. “subject_linkages” is a nested list organized on the hierarchy of subject > receiving cell type > linkages. Each subject’s slot in the list is populated with all linkages inferred by running dominoSignal on the cells from that subject. The character vector of intercellular linkages from the “rec_lig_cl” slot provide a binary representation of whether each possible interaction is on or off. The linkage summary also retains character vectors of intracellular linkages from the “clust_tf_rec” and all other signaling features stored in the linkages slot of each domino object. Though this is tailored to retrieving this information from a domino object, the Linkage Summary is agnostic to the method used to infer intercellular linkages. Thus, a Linkage Summary could be used to store and compare any inferred communication on the basis of cell type so long as the linkages of communication could be binarized as active or inactive. The Linkage Summary’s “subject_meta” slot contains a table listing the subjects with columns annotating variables describing the subjects that could be used for DCST comparisons. The Linkage Summary’s “subject_names” slot contains a factor vector of the names of subjects for easy access.

**Differential cell signaling test (DCST) functions**

The dominoSignal package also includes functions for tabulating the active linkages among subjects covered in the linkage summary (*count_linkages*) and conducting a DCST for a specified linkage type received by a shared cell type across independent variables annotated in the subject_meta table (*test_differential_linkages*). The *test_differential_linkages* function populates a contingency table for the linkage being tested where rows are the levels of the independent variable being tested and columns are the linkage being inferred as active or inactive. The resulting table, where each row corresponds to a tested linkage, provides the name of the linkage feature, the total number of subjects (total_n) and number of subjects with the active linkage (total_count), and columns for each level of the independent variable counting the number of subjects in the level (‘level’_n) and number of subjects with the active linkage (‘level’_count).

**Works Cited**

10X Genomics Datasets -Single Cell Gene Expression -Official 10x Genomics Support. *Support. Cell Gene ExpressionDatasets*.

Browaeys,R. *et al.* (2020) NicheNet: modeling intercellular communication by linking ligands to target genes. *Nat. Methods*, **17**, 159–162.

Cherry,C. *et al.* (2021) Computational reconstruction of the signalling networks surrounding implanted biomaterials from single-cell transcriptomics. *Nat. Biomed. Eng.*, **5**, 1228–1238.

Efremova,M. *et al.* (2020) CellPhoneDB: inferring cell–cell communication from combined expression of multi-subunit ligand–receptor complexes. *Nat. Protoc.*, **15**, 1484–1506.

Shao,X. *et al.* (2021) CellTalkDB: a manually curated database of ligand–receptor interactions in humans and mice. *Brief. Bioinform.*, **22**, bbaa269.

Su,J. *et al.* (2024) Cell–cell communication: new insights and clinical implications. *Signal Transduct. Target. Ther.*, **9**, 1–52.

Van de Sande,B. *et al.* (2020) A scalable SCENIC workflow for single-cell gene regulatory network analysis. *Nat. Protoc.*, **15**, 2247–2276.
