# Supplementary material for: Differential cell signaling testing for cell-cell communication inference from single-cell data by dominoSignal
Source: Bioinformatics. 2026 Feb 26;42(3):btag089. doi: 10.1093/bioinformatics/btag089 (PMC12998610; doi:10.1093/bioinformatics/btag089)
Supplement: btag089_Supplementary_Data [file btag089_supplementary_data.zip › Supplemental File 2.docx]

**Supplemental File 2: Signaling Simulation demonstrates sample sizes of at least 15 and cell types counts of at least 150 cells are sufficient for accurate identification of differential signals**

We analyzed cell signaling changes using simulated data with a known ground truth to benchmark the performance of our differential signaling test under varying conditions. The simulated data were designed to benchmark performance of both identification of true positive signaling differences and avoiding detection of false positives. Briefly, we simulated single-cell data measuring two cell types (A or B). Cells within each cell type were assigned a probability of expressing two possible ligands (L1 and L2) and two possible receptors (R1 and R2). L1 and R1 are part of a signaling pair capable of linkage as are L2 and R2. We simulated simplified intercellular signaling with two simulation conditions (C1 and C2) where L1 – R1 signaling from A to B ([B]:R1 <- L1:[A]) differentially occurred in C1, L1 – R1 autocrine signaling from B to B ([B]:R1 <- L1:[B]) differentially occurred in C2, and L2 – R2 signaling from A to B ([B]:R2 <- L2:[A]) occurred in both conditions (Figure 1B, Supplemental File 2 Figure 1A).

Linkage criteria were simplified to ligand and receptor expression above a threshold to maintain control of expected results. A linkage was active if 25% of the cells in the sender cell type, either A or B, expressed the linkage’s ligand and 25% of the cells in the receiver cell type expressed the linkage’s receptor. A full table of starting parameters for the number of cells of each type in the two conditions is available as Supplemental Table S1. Both R1 and R2 had a 30% probability of expression by cells in cluster B in C1 and in C2. [B]:R1 <- L1:[A] was programmed to be a differential linkage more likely to occur in C1 by L1 having a 30% probability of expression by cluster A cells in C1 but a 25% probability of expression by cluster A cells in C2. [B]:R1 <- L1:[B], the autocrine signal, was programmed to be more likely to occur in C2. Cells in Cluster B had a 10% chance of expressing L1 in C1 and a 30% chance of expressing L1 in C2. [A]:R2 <- L2:[B] served as a negative control, as the linkage was equally likely to occur in C1 or C2. Cells in cluster A had a 30% chance of expressing L2 in both conditions. The first two linkages, [B]:R1 <- L1:[A] and [B]:R1 <- L1:[B], were assessed as true positives. Occurrences of [A]:R2 <- L2:[B] being found differential were false positives.

To determine the number of samples sufficient to identify true positive differential signals between conditions, simulations were conducted varying the number of subjects in each condition from 5 to 65 in increments of 5. For each parameter set, 25 unique initializations of the simulation were conducted, and the number of initializations where each of the linkages was significantly differential (p < 0.05) was counted (Supplemental File Figure 1B, Supplemental Table S2). A sample size of 30 was required to consistently identify [B]:R1 <- L1:[A] as differential across all 25 initializations, though a sample size of 15 sufficed to correctly identify the differential linkage 23/25 times. The other differential linkage, [B]:R1 <- L1:[B], was correctly identified across all sample sizes. The relative robustness of [B]:R1 <- L1:[B] being correctly identified as differential compared to [B]:R1 <- L1:[A] is likely due to the difference in L1 expression probability across conditions. The probability of L1 expression by cells in cluster B differed by 0.2 between conditions while the difference in L1 expression probability by cells in cluster A was 0.1. The negative control [B]:R2 <- L2:[A] linkage was correctly identified as having an equal probability of occurring in conditions C1 and C2 across all initializations. Based on these simulations, we estimate a minimum of 15 samples are needed to accurately infer differential communication, suggesting that these methods are accurately employed in cohort level atlas studies or require an alternative approach to overcome small sample sizes that are more common for preclinical studies.

Another challenge in analysis of scRNA-seq data is accuracy of data derived from rare cell types representing a small number of cells profiled in a scRNA-seq data set. Low cell counts have been shown to impact accuracy of identifying differentially expressed genes (Squair *et al.*, 2021), and could have similar impacts on inferred cell communication derived from gene expression in these cell populations. To assess the impact of low cell numbers on identification of differential signaling, the number of cells in cluster B in condition C1 was varied from 50 cells to 500 cells in increments of 50 while maintaining consistent probability of ligand and receptor expression and comparing 20 subjects each from conditions C1 and C2 by DCST (Supplemental Table S3). The accuracy of identifying true positive differential linkages [B]:R1 <- L1:[A] and [B]:R1 <- L1:[B] was maintained across all cluster B sizes. However, the median p-value derived from testing the autocrine [B]:R1 <- L1:[B] linkage increased with lower cell numbers (Supplemental File 2 Figure 1C). The p-values were never high enough to fail to reject the null hypothesis that the proportion of simulated subjects with an active [B]:R1 <- L1:[B] linkage was equal between conditions C1 and C2. However, lower cell numbers raised p-values derived from the DCST (Supplemental File 2 Figure 1C). The [B]:R2 <- L2:[A] linkage programed to not be differential between conditions was prone to false rejections of the null hypothesis of equal proportions of active linkage in each condition at cell numbers of 150 and lower. This experiment demonstrates that rare cell populations with few cells represented in a scRNA-seq data set are at increased risk of false positive detection of differential signaling. These simulations suggest that results concerning receipt of signaling by cell types with cell numbers below 200 may be unreliable and must be accompanied by orthogonal experiments to validate signaling dependence on the tested variables in real datasets.


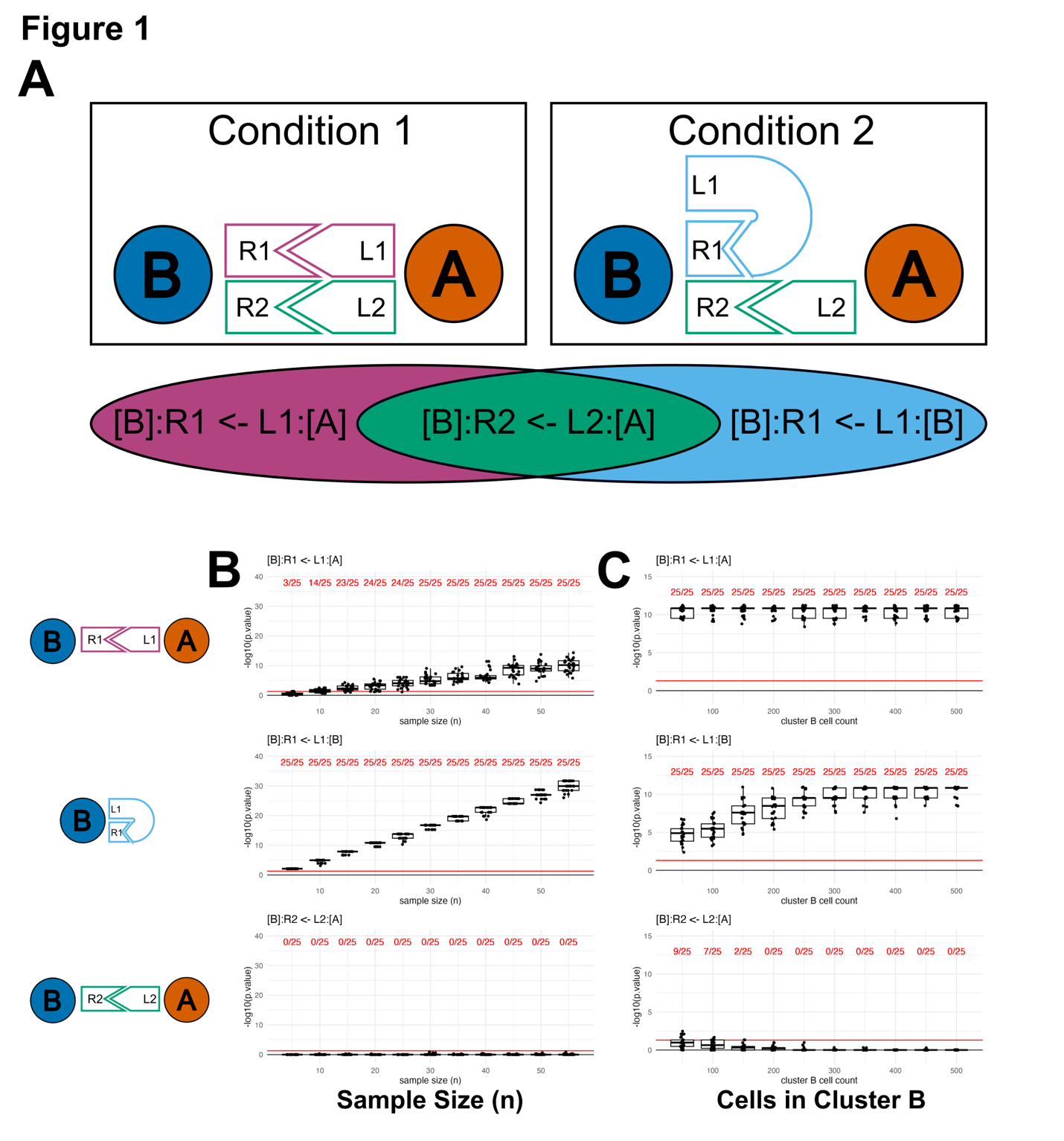


Figure 1: Assessment of performance of the differential cell signal testing in simulated data. (A) Graphical outline of signaling simulation. Cells belong to type A or B. Cells are simulated in two conditions with differing probabilities of signaling. Signaling occurs based on the percentage of cells expressing ligand-receptor pairs L1 to R1 and L2 to R2. The Venn diagram summarizes the interactions taking place exclusively in condition 1 (magenta), exclusively in condition 2 (blue), or equally likely in both conditions (green). Signals are phrased in terms of [“recipient cell”]:“receptor” <- “ligand”:[“sender cell”]. (B-C) P-values of differential signaling tests by Fisher’s Exact Test across repeated initializations of simulated data varying the number of subjects in conditions being compared (B) or number of cells belonging to type B (C). The tested intercellular signals are [B]:R1 <- L1:[A] (top), [B]:R1 <- L1:[B] (center), and [B]:R2 <- L2:[A] (bottom). The red line denotes a significance threshold of α = 0.05. The red proportion above denotes the number of unique initializations in which the tested achieved a p-value below 0.05.

**Methods for simulation of cell-cell communication and communication inference**

Simulated data were generated with predetermined intercellular linkages specified to be differential or consistent between conditions to have a ground truth against with to assess methods performance. The purpose of these simulations were to generate samples with expected intercellular linkages between two cell types. Ligand and receptor expression by cells was simplified to binary states of expressed (1) or not expressed (0) in each cell. The criterion for an intercellular linkage via these a ligand-receptor pairs was that at least 25% of the cells in the sender cell type express the ligand and that at least 25% of recipient cells express the receptor.

Two experimental conditions, C1 and C2, were designed for simulating cells for assessing intercellular linkages. Cells belonged to one of two cell types, A or B. Expression of two ligands, L1 and L2, and two receptors, R1 and R2, were simulated for each cell. L1-R1 and L2-R2 form pairs capable of intercellular linkage. When each cell was generated, the cell had a programmed probability of expressing each of the four signaling molecules based on a draw from a uniform distribution. These probabilities were specified based on the condition being simulated and the cell’s type. For example, a cell generated from cell type A in condition 1 had a 30% probability of expressing L1, 30% probability of expressing L2, 5% probability of expressing R1, and 5% probability of expressing R2. Probabilities of expression for each simulated cell type and condition under default parameters are listed in Supplemental Table S1. Upon generating all cells for a simulated sample, presence of intercellular linkages was assessed based on the intercellular linkage criteria stated above, and the result was stored as a Linkage Summary. Differential cell signaling between C1 and C2 was assessed by DCST using this Linkage Summary. The default parameters were designed so that the [B]:R1 <- L1:[A] linkage was more likely to occur in C1, [B]:R1 <- L1:[B] linkage was more likely to occur in C2, and [B]:R2 <- L2:[A] was equally likely to occur in each condition.

The effect of the number of subjects on correct inference of differential intercellular signaling was assessed by varying the number of subjects on identifying differential intercellular linkages for each condition from 5 subjects up to 65 subjects in increments of 5. The effect of cell number on correct inference of differential intercellular signaling was assessed by varying the number of cells in cell type B in condition C2 from 50 cells to 500 cells in increments of 50. The raw p-values derived from the DCST were compiled from 25 initializations of each parameter set and assessed for statistical significance at α = 0.05. Raw p-values were used as only 3 possible linkages were tested for each initialization.

**Works Cited**

Squair,J.W. *et al.* (2021) Confronting false discoveries in single-cell differential expression. *Nat. Commun.*, **12**, 5692.
