# Supplementary material for: Differential cell signaling testing for cell-cell communication inference from single-cell data by dominoSignal
Source: Bioinformatics. 2026 Feb 26;42(3):btag089. doi: 10.1093/bioinformatics/btag089 (PMC12998610; doi:10.1093/bioinformatics/btag089)
Supplement: btag089_Supplementary_Data [file btag089_supplementary_data.zip › Supplemental File 3.docx]

**Supplemental File 3: Detailed methods real-world single-cell datasets used for DCST testing**

**Single-cell RNA-seq data from human Pancreatic Ductal Adenocarcinoma tumors**

Single-cell RNA-seq data from human PDAC tumors were obtained from the datasets compiled and annotated as described in Guinn *et al.* (2024). In brief, this previous study integrated six data sets of scRNA-seq profiles from primary PDAC tumors (Bernard *et al.* 2019; Elyada *et al.* 2019; Peng *et al.* 2019; Lin *et al.* 2020; Moncada *et al.* 2020; Steele *et al.* 2020) and performed clustering to identify 11 cell types present in PDAC tumors (Guinn *et al.* 2024). In this current study, cells belonging to the fibroblast, epithelial cancer, and CD8 T cell types from the two largest data sets used by Guinn *et al.* (2024), 14,537 cells from Peng et al and 9,428 cells from Steele et al (Supplemental File 4 Figure 1A) were analyzed. These data were isolated into a Seurat object (Hao *et al.* 2021) and batch-corrected PCA embeddings for cells correcting for manuscript source were calculated using Harmony (Korsunsky *et al.* 2019) prior to UMAP visualization (Becht *et al.* 2019). Seurat module scores for gene sets associated with classical PDAC and basal-like PDAC as defined by Moffitt et al (Moffitt *et al.* 2015) were calculated for all epithelial cancer cells (Supplemental File 4 Figure 1B-C). Cells were typed as classical or basal based on which of the two module scores were greater in the cell, and samples were annotated as basal or classical if 50% or greater of their epithelial cancer cells were of that cancer cell type (Supplemental File 4 Figure 1D).

**Analysis of Visium spatial transcriptomics of pancreatic ductal adenocarcinoma**

Visium spatial transcriptomics data was obtained from a cohort of formalin-fixed and paraffin-embedded human surgical specimens containing pancreatic intraductal neoplasia proximal to PDAC tumors. Spatial transcriptomics profiling was performed using Visium spot-based capture (10X Genomics) as described in Bell et al (2022) and Lyman et al (2025). Data in GEO GSE254829 and GSE294669 was used and classical and basal labels were annotated as described in Bell et al (2022). Tissue section PDAC03 was selected for spatial validation of differential signaling identified in the human PDAC scRNA-seq data as the section had many spots containing PDAC (1696 PDAC spots). RNA Expression of *FGFR4* and *FGF2* normalized using the Seurat SCTransform function (v 4.1.1) was assessed for overlap with basal and classical regions of PDAC tissue and differential expression using the MAST test (Finak *et al.* 2015).

**Differential Cell Communication Testing on Panc02 Tumor Response to PancVAX and Immune Checkpoint Inhibitor Therapy**

Single-cell RNA-seq data of vaccine and immune checkpoint inhibitor treatment of a subcutaneous, murine model of PDAC generated from the Panc02 cell line are obtained from Huff et al (2023), and use the cellular annotations from that study (GEO GSE244992). In this current study, cells were subset to those belonging to the Untreated, PancVAX, and PancVAX + anti-PD-1 + anti-CTLA-4 treatment groups. TF activity score inference was conducted on each treatment group independently using SCENIC (v 0.11.0) (Aibar *et al.* 2017; Van de Sande *et al.* 2020) with mouse TF lists and TF motif references for the mm10 reference genome provided by cisTarget (Herrmann *et al.* 2012; Imrichová *et al.* 2015) (<https://resources.aertslab.org/cistarget/databases/>). 20 bootstraps were used for comparison of treatment groups. Ligand receptor pairs were sourced from CellTalkDB (v 1.0) (Shao *et al.* 2021), and possible interaction between ligand-receptor pairs *Cd80*-*Ctla4* and *Cd86*-*Ctla4* were manually added to the reference as they were not included in CellTalkDB v1.0 and are expected to be a target of anti-CTLA-4 therapy.

**scRNA and scATAC-seq profiling of MMTV-PyMT murine mammary tumors treated with Entinostat**

MMTV-PyMT mice, which develop mammary adenocarcinoma tumors due to expression of the *PyMT* transgene under an MMTV promoter, were used as a representative sample of mammary cancer with minimal cytotoxic immune cell infiltrates. At 80 days of age, female mice were randomized into assignment for treatment with Entinostat (ENT), an oral histone deacetylase inhibitor, delivered in mouse chow for a targeted oral intake of 12 mg/kg or assignment for untreated control (NT) receiving normal chow. After 14 days of treatment, mice were sacrificed, and tumors were harvested for enzymatic and mechanical digestion into single-cell suspensions. Whole tumor cell suspensions were profiled with scRNA-seq using 10x Genomics single cell Chromium library preparation. Both whole tumor and an immune-enriched single-cell suspension was created using magnetic bead selection for CD45+ cells and analyzed with single-cell ATAC-sequencing profiling using the single-cell combinatorial indexing assay for transposase accessible chromatin technology.

The scATAC-seq data were analyzed with Seurat (v 5.0.0) (Hao *et al.* 2024) and Signac (v 1.12.9004) (Stuart *et al.* 2021a) in R (v 4.3.1). Cells were retained if they had a nucleosome signal of less than 2 and a total number of peaks between 1000 and 8000 peaks. Cells with low transcription start site enrichment (< 1.5), high blacklist ratio (>0.04) and low fraction of reads in peaks (< 0.3) were excluded from analysis. Seurat’s *FindNeighbors* and *FindClusters* functions were used for clustering and data visualization on UMAP space. Cells were initially annotated as cancer associated fibroblasts (CAF), Neoplastic, or Immune based on chromatin accessibility and Signac GeneActivity scores (Stuart *et al.* 2021b). To further define subpopulations of immune cells, scRNA-seq and scATAC-seq were integrated using Seurat’s FindAnchors function for transfer learning (Butler *et al.* 2018; Stuart *et al.* 2021a). Cell population labels were transferred from scRNA-seq to the scATAC seq data using Canonical Correlation Analysis (CCA) (Stuart *et al.* 2021a). Based on the labels from the scRNA-seq data, the annotation of immune cells in the scATAC-seq data was able to be further refined into Lymphoid/NK and Myeloid cell types.

TF activity scoring based solely on RNA expression data was conducted using pySCENIC (v 0.11.0) (Aibar *et al.* 2017). The list of genes encoding transcription factors and rankings of TF binding motif enrichment within 500 bp and 10 kb windows of gene transcription start sites in the mouse mm10 reference genome were obtained from the cisTarget (Imrichová *et al.* 2015) resources website. Gene regulatory networks were learned from the raw RNA counts matrix using the grn function with method “GRNBoost.” Networks were pruned into TF regulons based on presence of TF binding motifs using the ctx function. TF activities were quantified in cells based on the learned regulons using the aucell function.

**Integration of scRNA-seq and scATAC-seq data in TF activity scoring by Targeted Regulons**

The Targeted Regulons method for integrating scATAC-seq chromatin accessibility data into the inference of TF activity scores was developed to improve score inference by pruning SCENIC regulons to only consider target genes with sufficient chromatin accessibility within a scored cell type. Chromatin accessibility is quantified based on the Signac gene activity score of the target gene (Stuart *et al.* 2021b). Target genes that have non-zero gene activity scores in at least 10% of cells belonging to the cell type in the scATAC-seq data are retained to form the cell type-specific regulons. The cell type-specific Targeted Regulons of TF to target gene associations are then used to score TF activity scores using AUCell (Aibar *et al.* 2017).

**DCST comparison between scRNA-seq analysis with SCENIC and integrated scRNA-seq and scATAC-seq data with Targeted Regulons**

TF activity scores were calculated for CAF, Lymphoid/NK, Myeloid and Neoplastic cells in the NT and ENT treatment groups were obtained from the RNA-seq data with SCENIC (v 0.11.0) and multi-omics data with Targeted Regulons as described above. To assess the effects of using SCENIC or Targeted Regulons on inference of intracellular linkages by dominoSignal, twenty bootstraps each were generated from profiles of cells in the NT treatment groups of scRNAseq profiles and underwent TF activity scoring with SCENIC using only RNA expression data or with Targeted Regulons using RNA expression and ATAC accessibility data. RNA expression data and TF activity scores were then used to infer cell-cell communication with dominoSignal, and the inferred intracellular signaling from receptors to TFs was compared using DCST (Supplemental File 8). For the TFs whose activity was inferred by both methods, intracellular linkages between these TFs and receptors were compared for all cell types by DCST using the bootstraps for SCENIC and Targeted Regulons. The number of intracellular linkages inferred between shared TFs for each cell type were plotted as a stacked barplot colored by whether the linkage was significantly differential in SCENIC, Targeted Regulons, or equally likely by either method. The overlap of inferred TF features for each method and treatment is shown as a Venn diagram generated using the ggvenn R package (v 0.1.10).

**Differential cell communication testing of effects of Entinostat on intercellular and intracellular communication in MMTV-PyMT tumors**

Twenty bootstraps each were generated from the scRNA-seq data and Targeted Regulons TF activity scores from the ENT and NT treatment groups. Intercellular linkages for all recipient cell types were compared using DCST. The leading differential intercellular signals received by Myeloid cells between NT and ENT treatment groups grouped by signals sent by Neoplastic cells or Lymphoid/NK cells were compared.

**Works Cited**

Aibar S, González-Blas CB, Moerman T *et al.* SCENIC: single-cell regulatory network inference and clustering. *Nat Methods* 2017;**14**:1083–6.

Becht E, McInnes L, Healy J *et al.* Dimensionality reduction for visualizing single-cell data using UMAP. *Nat Biotechnol* 2019;**37**:38–44.

Bell ATF, Mitchell JT, Kiemen AL *et al.* Spatial transcriptomics of FFPE pancreatic intraepithelial neoplasias reveals cellular and molecular alterations of progression to pancreatic ductal carcinoma. 2022:2022.07.16.500312.

Bernard V, Semaan A, Huang J *et al.* Single-Cell Transcriptomics of Pancreatic Cancer Precursors Demonstrates Epithelial and Microenvironmental Heterogeneity as an Early Event in Neoplastic Progression. *Clin Cancer Res* 2019;**25**:2194–205.

Butler A, Hoffman P, Smibert P *et al.* Integrating single-cell transcriptomic data across different conditions, technologies, and species. *Nat Biotechnol* 2018;**36**:411–20.

Elyada E, Bolisetty M, Laise P *et al.* Cross-Species Single-Cell Analysis of Pancreatic Ductal Adenocarcinoma Reveals Antigen-Presenting Cancer-Associated Fibroblasts. *Cancer Discov* 2019;**9**:1102–23.

Finak G, McDavid A, Yajima M *et al.* MAST: a flexible statistical framework for assessing transcriptional changes and characterizing heterogeneity in single-cell RNA sequencing data. *Genome Biol* 2015;**16**:278.

Guinn S, Kinny-Köster B, Tandurella JA *et al.* Transfer Learning Reveals Cancer-Associated Fibroblasts Are Associated with Epithelial–Mesenchymal Transition and Inflammation in Cancer Cells in Pancreatic Ductal Adenocarcinoma. *Cancer Res* 2024;**84**:1517–33.

Hao Y, Hao S, Andersen-Nissen E *et al.* Integrated analysis of multimodal single-cell data. *Cell* 2021;**184**:3573-3587.e29.

Hao Y, Stuart T, Kowalski MH *et al.* Dictionary learning for integrative, multimodal and scalable single-cell analysis. *Nat Biotechnol* 2024;**42**:293–304.

Herrmann C, Van de Sande B, Potier D *et al.* i-cisTarget: an integrative genomics method for the prediction of regulatory features and cis-regulatory modules. *Nucleic Acids Res* 2012;**40**:e114.

Huff AL, Longway G, Mitchell JT *et al.* CD4 T cell–activating neoantigens enhance personalized cancer vaccine efficacy. *JCI Insight* 2023;**8**, DOI: 10.1172/jci.insight.174027.

Imrichová H, Hulselmans G, Kalender Atak Z *et al.* i-cisTarget 2015 update: generalized cis-regulatory enrichment analysis in human, mouse and fly. *Nucleic Acids Res* 2015;**43**:W57–64.

Korsunsky I, Millard N, Fan J *et al.* Fast, sensitive and accurate integration of single-cell data with Harmony. *Nat Methods* 2019;**16**:1289–96.

Lin W, Noel P, Borazanci EH *et al.* Single-cell transcriptome analysis of tumor and stromal compartments of pancreatic ductal adenocarcinoma primary tumors and metastatic lesions. *Genome Med* 2020;**12**:80.

Lyman MR, Mitchell JT, Raghavan S *et al.* Spatial proteomics and transcriptomics reveal early immune cell organization in pancreatic intraepithelial neoplasia. *JCI Insight* 2025;**10**, DOI: 10.1172/jci.insight.191595.

Moffitt RA, Marayati R, Flate EL *et al.* Virtual microdissection identifies distinct tumor- and stroma-specific subtypes of pancreatic ductal adenocarcinoma. *Nat Genet* 2015;**47**:1168–78.

Moncada R, Barkley D, Wagner F *et al.* Integrating microarray-based spatial transcriptomics and single-cell RNA-seq reveals tissue architecture in pancreatic ductal adenocarcinomas. *Nat Biotechnol* 2020;**38**:333–42.

Peng J, Sun B-F, Chen C-Y *et al.* Single-cell RNA-seq highlights intra-tumoral heterogeneity and malignant progression in pancreatic ductal adenocarcinoma. *Cell Res* 2019;**29**:725–38.

Shao X, Liao J, Li C *et al.* CellTalkDB: a manually curated database of ligand–receptor interactions in humans and mice. *Brief Bioinform* 2021;**22**:bbaa269.

Steele NG, Carpenter ES, Kemp SB *et al.* Multimodal mapping of the tumor and peripheral blood immune landscape in human pancreatic cancer. *Nat Cancer* 2020;**1**:1097–112.

Stuart T, Srivastava A, Madad S *et al.* Single-cell chromatin state analysis with Signac. *Nat Methods* 2021a;**18**:1333–41.

Stuart T, Srivastava A, Madad S *et al.* Single-cell chromatin state analysis with Signac. *Nat Methods* 2021b;**18**:1333–41.

Van de Sande B, Flerin C, Davie K *et al.* A scalable SCENIC workflow for single-cell gene regulatory network analysis. *Nat Protoc* 2020;**15**:2247–76.
