# Supplementary material for: Differential cell signaling testing for cell-cell communication inference from single-cell data by dominoSignal
Source: Bioinformatics. 2026 Feb 26;42(3):btag089. doi: 10.1093/bioinformatics/btag089 (PMC12998610; doi:10.1093/bioinformatics/btag089)
Supplement: btag089_Supplementary_Data [file btag089_supplementary_data.zip › Supplemental File 4.docx]

**Supplemental File 4: Annotation of pancreatic ductal adenocarcinoma compendium samples as Classical and Basal predominant**

**
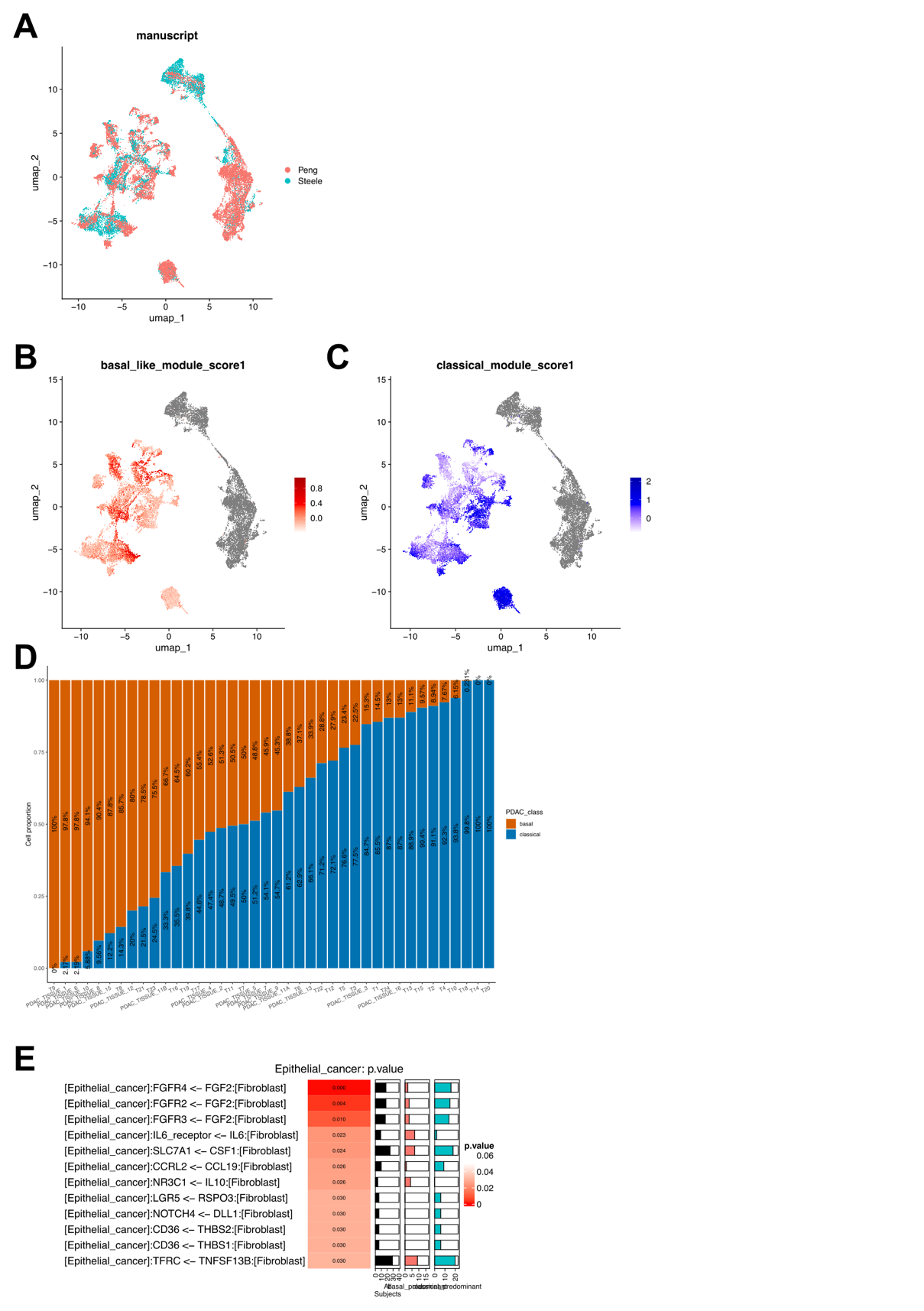
**

**Figure 1: Annotation of samples from PDAC compendium as basal or classical predominant.** (A-C) UMAP plots of PDAC tumor cells scRNA-seq profiles from 40 subjects annotated by manuscript data source (A), basal module score (B), and classical module score (C). (D) Stacked barpot of samples colored by the percentage of epithelial cancer cells typed as basal (orange) or classical (blue). Bars are overlayed with percentage of cells belonging to the group. (E) Top 12 Differential intercellular signals between basal (orange) and classical (blue) subjects with raw p-values less than 0.05.
