# Supplementary material for: Differential cell signaling testing for cell-cell communication inference from single-cell data by dominoSignal
Source: Bioinformatics. 2026 Feb 26;42(3):btag089. doi: 10.1093/bioinformatics/btag089 (PMC12998610; doi:10.1093/bioinformatics/btag089)
Supplement: btag089_Supplementary_Data [file btag089_supplementary_data.zip › Supplemental File 5.docx]

**Supplemental File 5: Bootstrapping enables robust detection of differential intercellular signaling in scRNA-seq data without biological replicates**

The simulation studies and analysis of the human PDAC cohort demonstrate the use of DCST on scRNA-seq datasets containing multiple samples in each condition to be compared. Some studies limit sample sizes or pool biological replicates for cost-effective hypothesis-generation scRNA-seq studies. To extend the applicability of the DCST to small data sets lacking sufficient biological replicates to apply the Fisher test to compare between groups of samples, we developed an approach to simulate biological replicates through bootstrapping of cells. For each cell type in a data set, cells are sampled with replacement until the number of sampled cells matches the number of cells from that type in the parent sample, generating a bootstrap. Each generated bootstrap is then subjected to CCCI, compiling the inferred intercellular and intracellular linkages within the Linkage Summary format to facilitate use of DCST (Supplemental File 5 Figure 1A).

We demonstrated this bootstrapping approach to DCST using a scRNA-seq data set designed to compare changes in the microenvironment between different therapeutic conditions. Briefly, we used scRNA-seq data from Huff et al (2023) that compared the tumor microenvironment changes from mice bearing subcutaneous tumors from the Panc02 cell line (Corbett *et al.*, 1984; Partecke *et al.*, 2011) treated with either vehicle control (Untreated), a personalized neoantigen peptide vaccine (termed ‘PancVAX’), or PancVAX in combination with the immune checkpoint inhibitors anti-PD-1 and anti-CTLA-4 (PancVAX + anti-PD-1 + anti-CTLA-4) (Supplemental File 5 Figure 1B). Profiling of these murine tumors revealed distinct expression profiles for multiple cell types critical to the tumor immune response including CD8+ T cells, CD4+ T cells, natural killer cells, B cells, macrophages, monocytes, myeloid suppressor cells, dendritic cells, endothelial cells, and cancer cells (Supplemental File 5 Figure 1C).

We first sought to leverage simulations generated by distinct sampling of this dataset to benchmark performance of this bootstrapping approach prior to demonstrating the biological findings from these analyses. To ensure that bootstrapping consistency of inferred signaling, eight unique initializations of bootstrapping subjects each from the Untreated and PancVAX groups were conducted and subjected to DCST comparing the intercellular linkages received by each of the represented cell types. The number of initializations out of eight where each intercellular linkage was differential between Untreated and PancVAX was assessed (Supplemental File 5 Figure 1D). For each cell type, at least half of the tested interactions were identified as differential, based on an FDR-adjusted p-value less than 0.05, in most of the initializations (>= 5/8 initializations). Among tested intercellular linkages received by tumor associated macrophages that were identified as differential in at least one initialization (Supplemental File 5 Figure 1E), incoming linkages that occurred in all eight initializations were by far the most frequent (478/1346 tested linkages), though linkages identified as differential in single initializations were the second most frequent (217/1346 tested linkages). Using the same approach to assess robustness of received intercellular linkages in all cell types (Supplemental File 5 Figure 2), a majority of intercellular linkages identified as differential were robust across unique initializations of bootstrap sampling. However, cell types represented by very few cells, such as endothelial cells in this data set, have less consistency in the number of differential intercellular linkages across initializations. The occurrence of linkages found differential in a single initialization illustrates the risk of bootstrapping to overrepresent or underrepresent subpopulations of cells within a cell type that may be significant contributors to a CCCI method’s criteria for intercellular linkage.

Variation generated through the bootstrapping approach could produce spurious identification of differential linkages caused by chance sampling of cells. We therefore tested if two unique initializations of bootstrapping from the same data set would result in any signals being identified as statistically differential by the DCST, despite being derived from the same parent data set. Differential signaling simulations illustrated the importance of DCST comparisons including at least 15 subjects in each group being compared (Supplemental File 1 Figure 1B). This established our basis that any bootstrapping from real data would use at least 15 bootstraps for each condition. Differential intercellular linkages were tested between two unique initializations bootstrapping from the PancVAX group (Supplemental File 5 Figure 3). No incoming intercellular linkages were found to be significantly differential between the two bootstrapping initializations from the same data. This demonstrates that variation generated through bootstrapping is not sufficient to cause false detection of differential linkages provided the number of bootstraps is large enough.


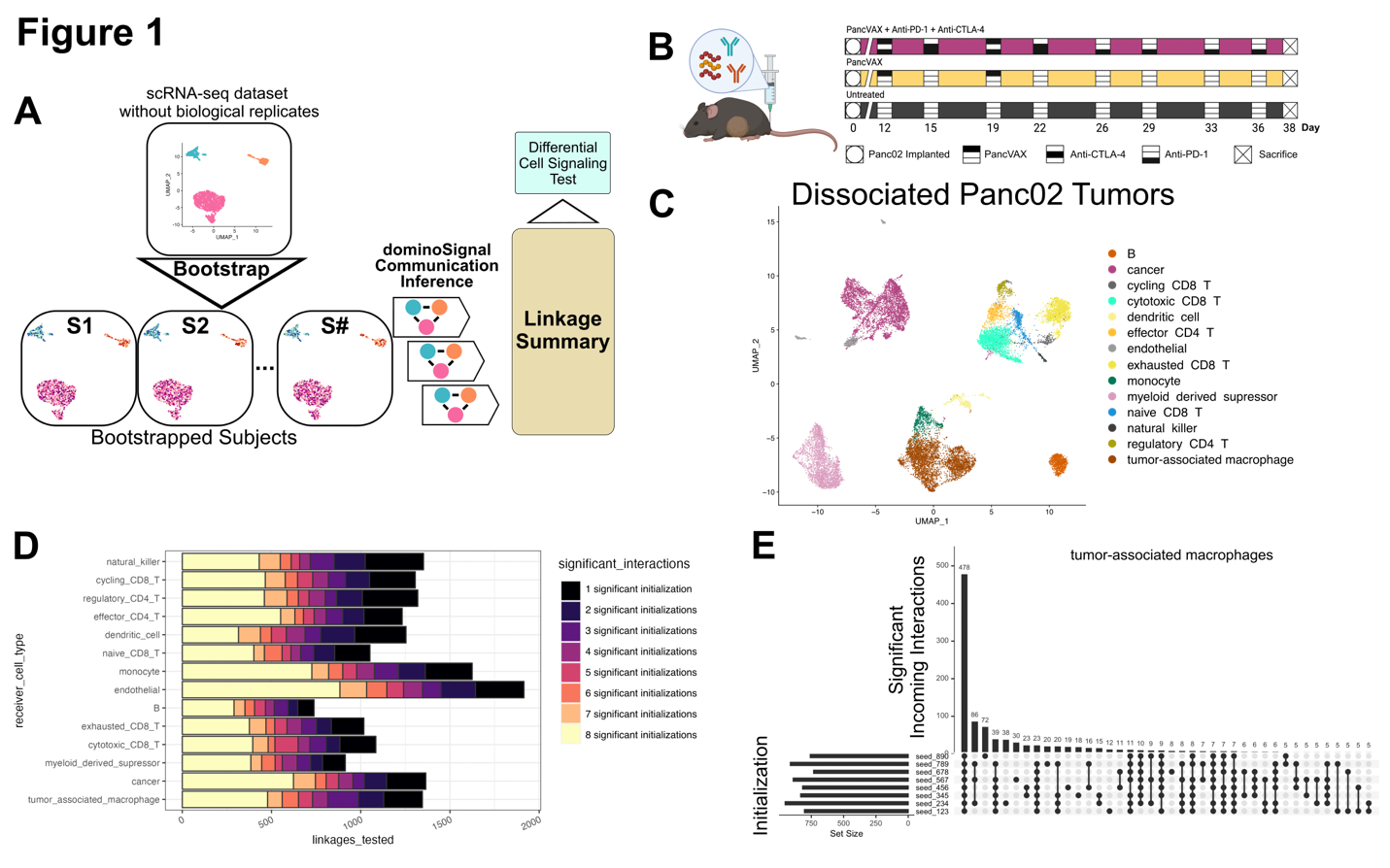


**Figure 1: Assessing differential signaling in datasets without biological replicates through bootstrapping**. (A) Graphical outline of bootstrapping approach in which cells are sampled with replacement from each cell type present in a subject until as many cells of that type from the original data set are obtained. Each bootstrapped sample is treated as an individual subject within the groups being compared via the differential signaling test. (B) Treatment regimen of mice which were implanted with Panc02 tumors and treated with PancVAX neoantigen cancer vaccine (gold), PancVAX in combination with anti-PD-1 and anti-CTLA-4 immune checkpoint inhibitors (magenta), or untreated given only isotype control (grey). (C) UMAP plot of scRNA-seq data of treated Panc02 tumors from Huff *et al.* (2023)that is used as the basis of our bootstrapped DCST analysis. (D) Robustness of differential signals received by cell types between untreated and PancVAX-treated mice across eight unique initializations of bootstrapping. Bar plots display the number of tested linkages found to be differential in all eight initializations (yellow) down to those differential in only one initialization (black). (E) Upset plot counting the occurrence differential signals received by tumor-associated macrophages across the eight initializations.


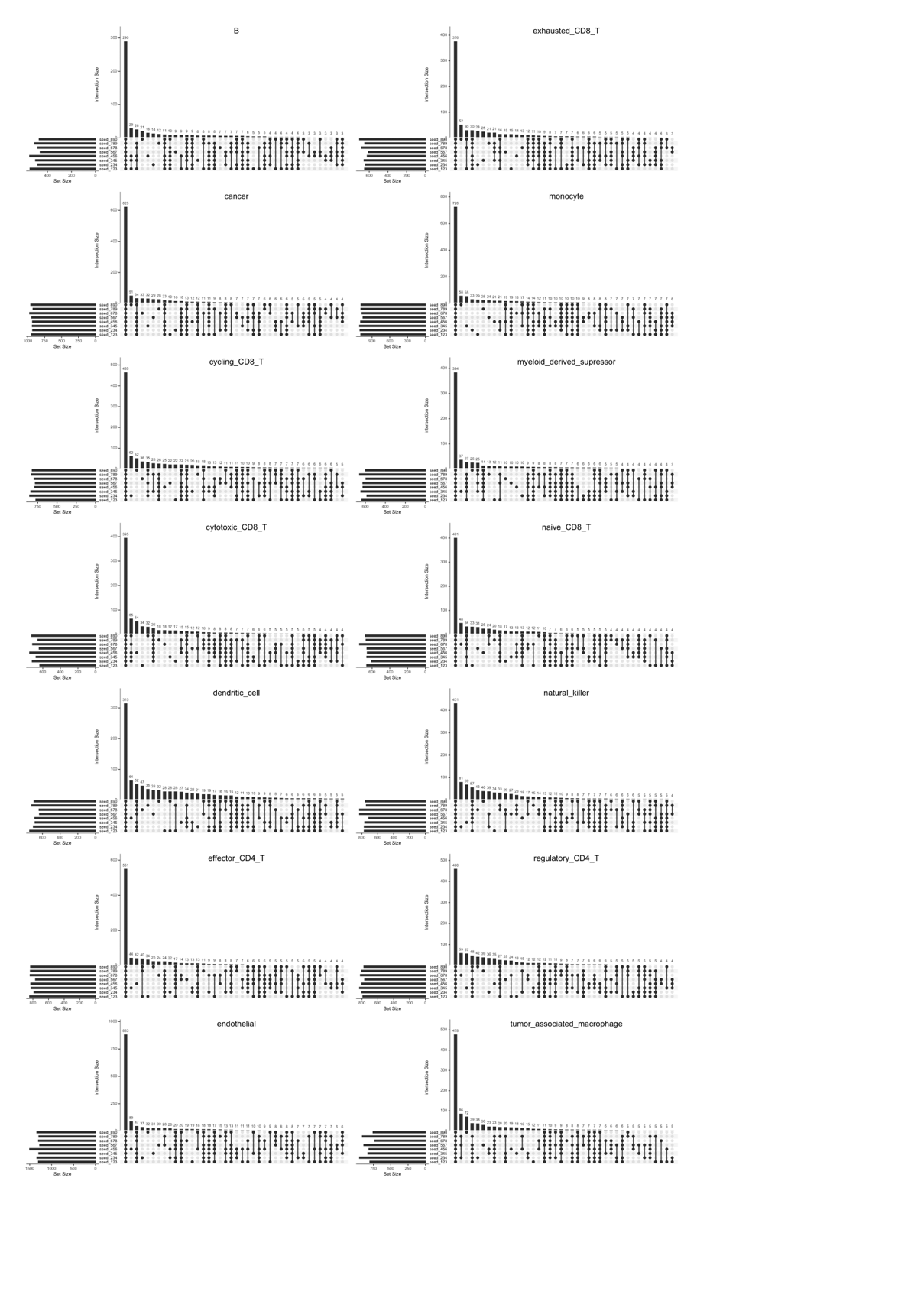


**Figure 2: Upset plots of the occurrence differential signals received cell types in Panc02 tumors across 8 initializations of comparing bootstraps from PancVAX and PancVAX + anti-PD-1 + anti-CTLA-4 treatment groups**. Receiving cell types include B cells, cancer, cycling CD8 T cells, cytotoxic CD8 T cells, dendritic cells, effector CD4 T cells, endothelial cells, exhausted CD8 T cells, monocytes, myeloid-derived suppressor cells, naïve CD8 T cells, natural killer cells, regulatory CD4 T cells, and tumor-associated macrophages

**Methods for bootstrapping of sample replicates for DCST analysis of pooled scRNA-seq data**

In the cases of assessing DCST in scRNA-seq data sets without annotation of source subjects as biological replicates, variation representative of biological replicates was generated by bootstrapping. To bootstrap, the scRNA-seq data set was separated into the two groups to be compared by DSCT. For each bootstrap from a group, cells were generated on a per cell type basis by uniform sampling with replacement, as implemented by the sample function in the base package in R (v 4.2.0). Sampling took place until as many cells of the type were drawn as were present in the original group. For CCCI by dominoSignal in bootstrapped data, cells in the bootstrap had the same TF activity scores, raw RNA counts, and normalized RNA expression as the corresponding cells from which they were sampled. RNA expression scaling was conducted on the bootstrapped normalized RNA expression once all sample draws were complete. CCCI with dominoSignal on each bootstrap proceeded using parameters as described in ‘Parameters for dominoSignal cell-cell communication inference on real data sets’. Linkages inferred by dominoSignal were stored as a Linkage Summary where each bootstrap was treated as a unique subject and annotated with the condition of the original data used to generate the bootstrap in the “subject_meta” table.

**Multi-level DCST for variance in probability of signaling across more than two levels**

The implementation of the Fisher’s Exact Test within DCST allows for testing of contingency tables with more two levels of the considered grouping variable. In the case of grouping samples in DCST with a variable that has more than two levels, the DCST tests the null hypothesis that the probability of a signal being active is equal across samples of all levels. We demonstrate this application of DCST with dominoSignal by testing for intracellular signaling incoming to exhausted CD8 T cells that differ across the Untreated, PancVAX, and PancVAX + anti-PD-1 + anti-CTLA-4 treatment groups (Supplemental File 5 Figure 3). Testing 20 bootstraps from each treatment, the leading edge of incoming signals from tumor-associated macrophages includes *Il10* signaling to *Il10rb* as an immunosuppressive signal present in all bootstraps from the Untreated and PancVAX bootstraps but completely in PancVAX + anti-PD-1 + anti-CTLA-4 samples where the immune checkpoint blockade works to maintain cytotoxic T cell function.


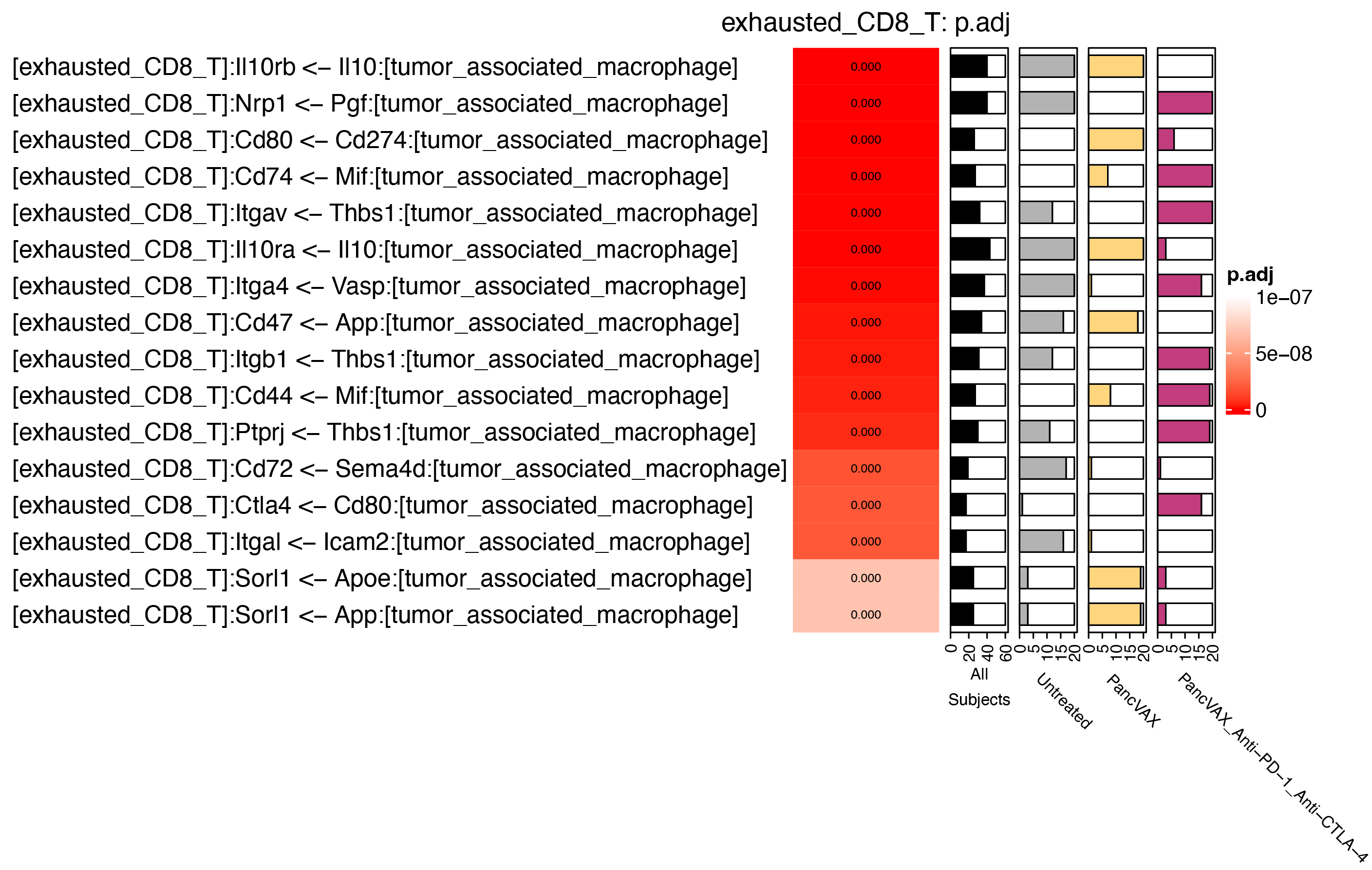


**Figure 3: Multi-level DCST of intercellular signaling received by exhausted CD8 T cells in PancVAX treatment**. Intercellular signals received by exhausted CD8 T cells from tumor associated macrophages whose probability of occurring vary significantly across bootstraps from Untreated, PancVAX, and PancVAX + anti-PD-1 + anti-CTLA-4 treatment groups. Signals with an FDR-adjusted p-value less than 1 * 10^-7^ are displayed. Gradient values correspond to FDR-adjusted p-value. Proportion bars display the number of bootstrapped subjects with active signaling in all subjects (black), Untreated (grey), PancVAX (gold), or PancVAX + anti-PD-1 + anti-CTLA-4 (magenta).

Works Cited

Corbett,T.H. *et al.* (1984) Induction and Chemotherapeutic Response of Two Transplantable Ductal Adenocarcinomas of the Pancreas in C57BL/6 Mice1. *Cancer Res.*, **44**, 717–726.

Datlinger,P. *et al.* (2021) Ultra-high-throughput single-cell RNA sequencing and perturbation screening with combinatorial fluidic indexing. *Nat. Methods*, **18**, 635–642.

Huff,A.L. *et al.* (2023) CD4 T cell–activating neoantigens enhance personalized cancer vaccine efficacy. *JCI Insight*, **8**.

McGinnis,C.S. *et al.* (2019) MULTI-seq: sample multiplexing for single-cell RNA sequencing using lipid-tagged indices. *Nat. Methods*, **16**, 619–626.

Partecke,L.I. *et al.* (2011) A Syngeneic Orthotopic Murine Model of Pancreatic Adenocarcinoma in the C57/BL6 Mouse Using the Panc02 and 6606PDA Cell Lines. *Eur. Surg. Res.*, **47**, 98–107.

Stoeckius,M. *et al.* (2018) Cell Hashing with barcoded antibodies enables multiplexing and doublet detection for single cell genomics. *Genome Biol.*, **19**, 224.
