# Supplementary material for: Differential cell signaling testing for cell-cell communication inference from single-cell data by dominoSignal
Source: Bioinformatics. 2026 Feb 26;42(3):btag089. doi: 10.1093/bioinformatics/btag089 (PMC12998610; doi:10.1093/bioinformatics/btag089)
Supplement: btag089_Supplementary_Data [file btag089_supplementary_data.zip › Supplemental File 6.docx]

**Supplemental File 6: Annotation of cell types from MMTV-PyMT tumors**


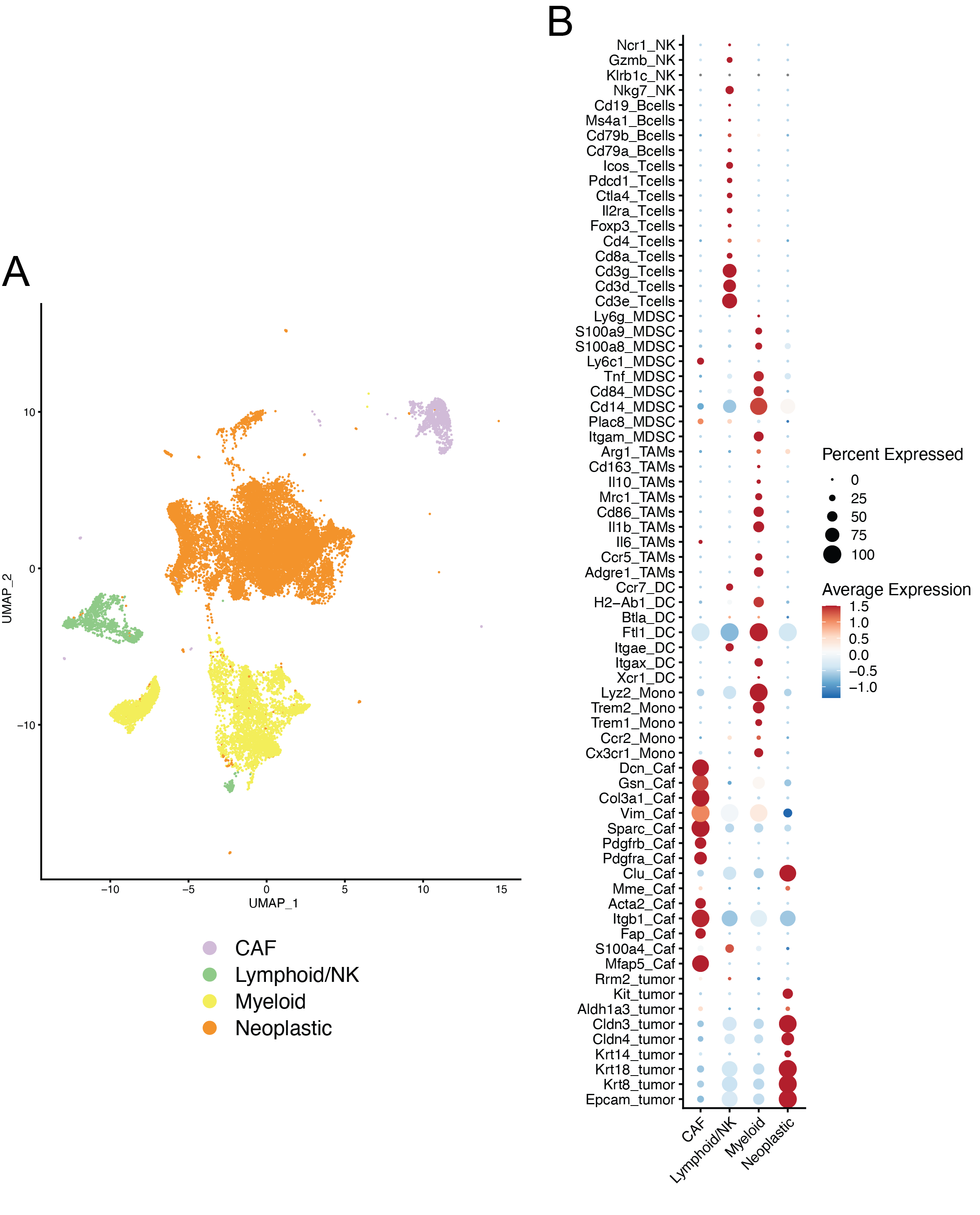


**Figure 1: Cluster annotation and marker gene expression in MMTV-PyMT cell types.** (A) UMAP plot of cells from MMTV-PyMT tumors colored based on annotated cell type as cancer-associated fibroblast (CAF, purple), Lymphoid/NK (green), Myeloid (yellow), or Neoplastic (orange). (B) Dot plot of marker gene expression in each annotated cell type. Dot size scales with the precent of cells in the cell type that express the gene, and dot color scales with average expression of the gene among cells of that type. Columns represent each cell type and rows are annotated with the gene expressed (left) and their characteristic cell type (right).
